# A resource for integrated genomic analysis of the human liver

**DOI:** 10.1038/s41598-022-18506-z

**Published:** 2022-09-07

**Authors:** Yi-Hui Zhou, Paul J. Gallins, Amy S. Etheridge, Dereje Jima, Elizabeth Scholl, Fred A. Wright, Federico Innocenti

**Affiliations:** 1 Department of Biological Sciences, North Carolina State University, Raleigh NC State University, Raleigh, NC 27695 USA; 2 Bioinformatics Research Center, North Carolina State University, Raleigh NC State University, Raleigh, NC 27695 USA; 3 Division of Pharmacotherapy and Experimental Therapeutics, UNC Eshelman School of Pharmacy, University of North Carolina, Chapel Hill, NC 27599 USA; 4 Department of Statistics, North Carolina State University, Raleigh NC State University, Raleigh, NC 27695 USA

**Keywords:** RNA sequencing, Genetics research, Bioinformatics

## Abstract

In this study, we generated whole-transcriptome RNA-Seq from *n* = 192 genotyped liver samples and used these data with existing data from the GTEx Project (RNA-Seq) and previous liver eQTL (microarray) studies to create an enhanced transcriptomic sequence resource in the human liver. Analyses of genotype-expression associations show pronounced enrichment of associations with genes of drug response. The associations are primarily consistent across the two RNA-Seq datasets, with some modest variation, indicating the importance of obtaining multiple datasets to produce a robust resource. We further used an empirical Bayesian model to compare eQTL patterns in liver and an additional 20 GTEx tissues, finding that MHC genes, and especially class II genes, are enriched for liver-specific eQTL patterns. To illustrate the utility of the resource to augment GWAS analysis with small sample sizes, we developed a novel meta-analysis technique to combine several liver eQTL data sources. We also illustrate its application using a transcriptome-enhanced re-analysis of a study of neutropenia in pancreatic cancer patients. The associations of genotype with liver expression, including splice variation and its genetic associations, are made available in a searchable genome browser.

## Introduction

Genomic studies of the human liver have predominantly focused on its role as the most important organ for detoxification^1^, while recent research has also expanded an understanding of its role in metabolism and homeostasis^2^, and an important role in immunology^3^. Studies of genetic influence on liver-related phenotypes have elucidated predisposing factors to liver disease^4^, drug-induced liver injury^5^, and have provided evidence for pharmacogenomic variation in response to chemotherapy^6^ and other drugs^7,8^. However, relevant human genome-wide association studies (GWAS), especially for pharmacogenomics, have been challenging due to sample size limitations^9^. Furthermore, many of these analyses have been discovery-based, while an improved mechanistic understanding of genetic regulation may be needed in order to identify novel targets and develop novel therapeutics^10^.

The utility of genetic association for liver-relevant phenotypes has also been shown less directly. Drugs that exploit targets the genes of which have evidence of GWAS association for multiple phenotypes have been shown to have greater success in early phase clinical trials^11^, following work by Nelson et al.^12^, who used liver expression quantitative trait loci (eQTLs) as a key source of evidence for genetic and physiological relevance. Here eQTLs serve as a bridge between genetic association and phenotypes, likely reflecting causal genetic effects^13^ and the more proximal effects of gene expression as intermediate traits^14^.

Liver eQTL studies using microarray expression quantification have been among the largest performed (after blood)^15^, and a recent liver eQTL meta-analysis^16^ in *n* = 1183 individuals described evidence of sex-biased effects and colocalization for loci related to blood metabolite and lipid levels. These previous liver eQTL studies provided important results, but have encountered some limitations, partly due to sensitivity and specificity issues common with microarrays^17^. In addition, previous microarray-based eQTL studies have not enabled study of splicing variation^18^ under genetic influence. RNA sequence-based genotype-expression resources such as the Genotype-Tissue Expression project (GTEx)^19^ have greatly advanced our understanding of genetic variation in gene regulation (including splicing) across a variety of human tissues, including liver. However, limitations in sample size and in representing potential heterogeneity in sampling protocols has prevented the community from fully exploiting the connections between tissue-specific variation and human disease. In addition, the focus of GTEx has emphasized functional information and annotation for the eQTLs (i.e., associated SNPs)^20^, leaving considerable room for analysis of the genes that are eQTL targets within a tissue of interest. In contrast, a perspective based on the target genes can potentially provide a more immediately interpretable context, with the ability to reach clear conclusions on aspects such as pathway enrichment, and in quantifying degree of tissue specificity among gene targets of eQTLs. Moreover, in contrast to earlier GTEx papers^21^, which had employed fully Bayesian methods to quantify tissue specificity among eQTLs, the final GTEX v8^22^ has provided less formal modeling of tissue-tissue relationships, leaving room to expand our understanding of these relationships.

The extent of heterogeneity across eQTL studies has been relatively unexplored, including for liver. Many previous studies had employed microarrays, so that the sources of observed variation across studies are difficult to attribute definitively, for example to heterogeneity in sampling protocols vs. platform variation. A potential strategy to identify meaningful heterogeneity across eQTL studies is to examine variation in comparison to phenomena such as the degree of enrichment of eQTLs with respect to GWAS signals^23^. Moreover, for completeness, eQTL association analyses should not be restricted to a single analysis type, but should include both robust association methods^24^ and the newer concept of quantification of allelic fold-change^25,26^.

In this study, we performed RNA-Seq profiling of *n* = 192 liver samples (hereafter the “UNC Liver” samples), nearly doubling the number (*n* = 208) of liver samples available from the GTEx project^19,22^. We present (i) new data with (ii) a re-analysis of GTEx liver data, and analyses including 19 additional GTEx tissues, and (iii) results from the microarray datasets comprising a previous liver eQTL meta-analysis^16^, in order to create a more comprehensive resource of liver eQTL results. Our analyses include results from robust association methods^24,27^, quantification of allelic fold-change^25,26^, and exon usage-based evidence for splicing eQTLs^28^. This comprehensive work has created the largest and most complete liver eQTL resource with this requisite depth of analysis. We provide novel enrichment observations, and careful modeling to quantify the degree of liver specificity in comparison to other tissues. In addition, we illustrate the utility of the data by describing a novel method to combine transcriptome-wide association results across our datasets, and illustrate the approach with an application to genetic association with treatment-associated neutropenia in pancreatic cancer patients. We present *cis*-eQTL results in a new public portal, with the link listed in https://github.com/zhouLabNCSU/Liver_project_resources.

## Results

### Data sources and quality control

The UNC Liver (RNA-Seq) data have not been previously described for eQTL analysis, although a minority subset of the patient samples overlap with the previous microarray meta-analysis^16^. Samples were subjected to extensive quality control, emphasizing appropriate matching of genotype and expression samples (Methods), reducing the number of paired genotype-expression samples from *n* = 206 to *n* = 192. Analyses were performed using expression of 29,245 genes and approximately 10.8 million genotyped or imputed single nucleotide polymorphisms (SNPs, see Methods). Data for 20 tissues including GTEx liver (*n* = 208) from the GTEx project version 8^22^ (RNA-Seq and genotypes) were obtained from dbGaP phs000424.v1.p1. This set of tissues had been previously analyzed using GTEx v6 data by Li et al.^29^. Finally, results from a previous meta-analysis of four individual microarray liver eQTL studies^16^ were used for comparison for some analyses. The sample sizes, number of genes, and other details for all data sources are shown with publication links in Supplementary Table 1.

### Association results

For the GTEx liver and UNC Liver datasets (RNA-Seq) local eQTL analysis (*cis,* ± 1 Mb window from the transcription start site), Matrix-eQTL^24^ was used for primary linear modeling after a robust expression transformation as used in GTEx , and ACMEeQTL^26^ as an allelic fold-change alternate approach. Distant (*trans*) association mapping was performed using Matrix-eQTL and vetting to avoid spurious cross-mapping as described in Methods. The four datasets and combined meta-analysis from^16^ were used as published. An “eGene” analysis based on the FastQTL software^27^ was used to generate gene-level *p* values for the RNA-Seq liver datasets.

**Figure 1:**
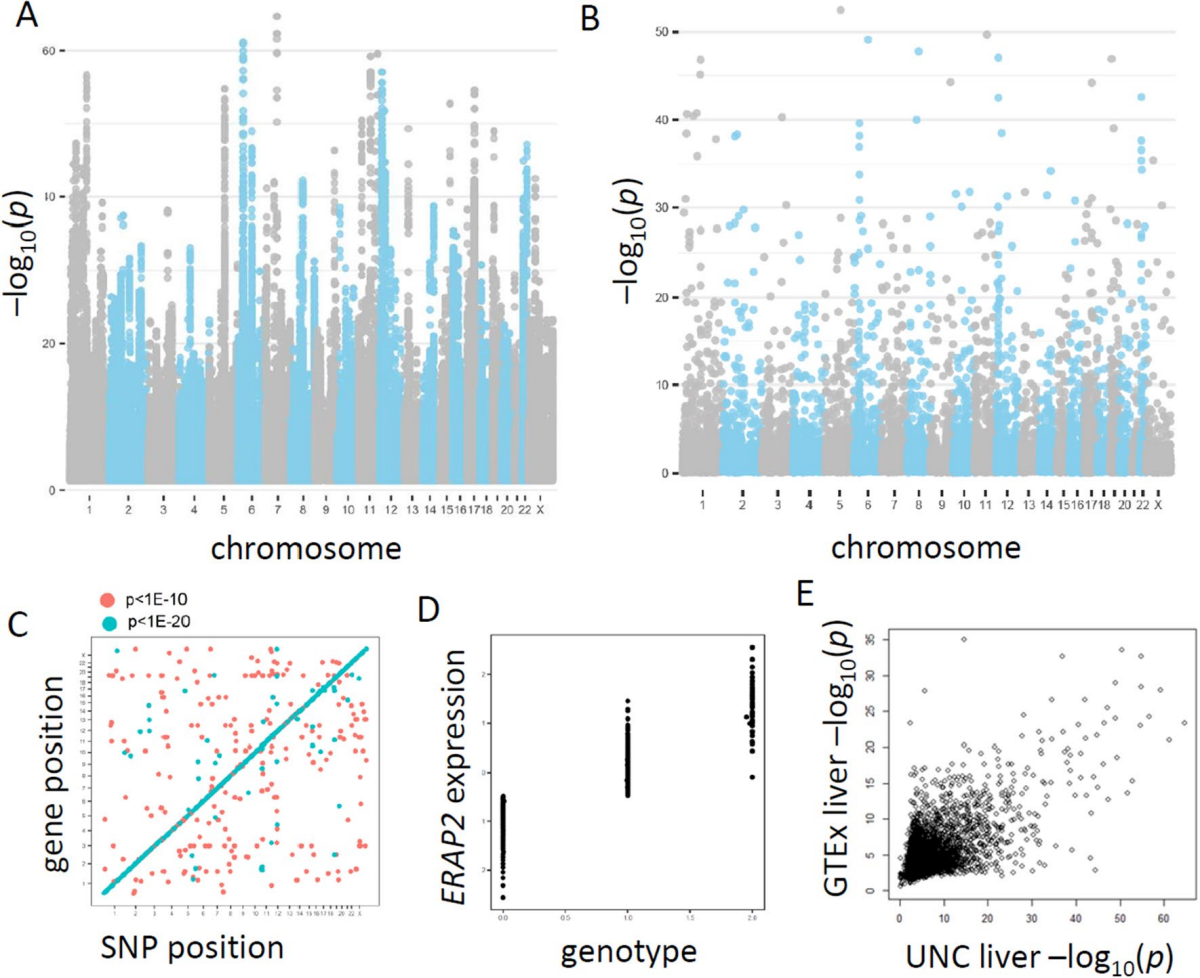
Liver RNA-Seq eQTLs. (**A**) SNP-level analysis of the UNC liver data, showing minimum association *p* values for local (*cis*) SNPs for each gene. (**B**) Gene level (eGene) results for the resource. (**C**) Transcriptome-wide association plot, showing SNP genomic position on the x-axis, and transcription start site for the gene on the y-axis. Association *P* values are color-coded according to two levels of stringency as shown. (**D**) Normalized expression vs. genotype for a strongly associated gene and SNP rs2910788, expressed as number of minor alleles (association *p* < 10^–20^). (**E**) *P* values for an allelic fold-change measure show correspondence of gene-level results for GTEx liver vs. the UNC liver data (Spearman ρ = 0.30, *p* < 0.001).

Figure 1A shows a Manhattan plot from the linear regression analysis for the UNC Liver data, using the minimum *p* value per gene for *cis*-eQTLs, while Fig. 1B shows the analogous results for the eGene^27^ analysis, for which *p* values represent gene-level significance. A total of 881,173 SNP*gene eQTL findings were significant at false discovery *q* < 0.1, for 18,051 unique genes, and 6748 eGenes. For allelic fold change in UNC Liver, there were 615,195 significant eQTL findings at *q* < 0.1, representing 15,407 unique genes. The top-ranked genes for the two analyses are shown in a higher-resolution version in Supplementary Figs. 1 and 2. Gene-level results, including the most significantly associated SNP for gene, for these and subsequent analysis methods are provided in Supplementary File 1. Figure 1C shows a transcriptome-wide eQTL plot for the UNC Liver data, with *cis*-associations generally much more strongly significant than *trans*-associations. The GTEx Consortium found no significant liver *trans*-eQTLs after applying strict criteria to avoid spurious cross-mapping^22^, while after applying similar criteria to UNC Liver we found five significant *trans*-eQTLs, targeting *FMO2*, *SRP54* (two different *trans*-eQTLs), *SKA3*, and *CLCN5*. These findings and a catalog of *trans*-eQTL findings for the two liver RNA-Seq datasets are shown in Supplementary Table 2, with mapping quality designations. Figure 1D shows genotype (number of minor alleles) for SNP rs2910788 vs. normalized expression for *ERAP2*, the most significant gene in the eGene analysis. Comparison of the UNC Liver data to GTEx liver for the allelic fold-change *p* values in Fig. 1E shows broad concordance of results across the two studies.

### Enrichment

**Figure 2:**
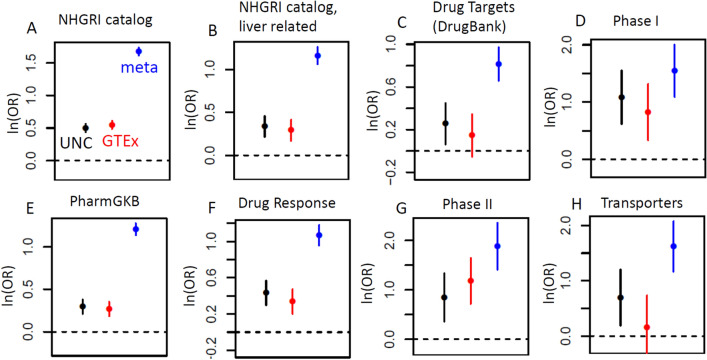
Enrichment of disease- and pharmacologically-relevant genes among liver eQTLs, as described in text, for UNC liver, GTEx liver, and the microarray meta-analysis. Natural logarithms of odds ratios (95% confidence intervals) are shown for enrichment of genes with cis-eQTLs with *p* < 10^–5^ and each corresponding gene set described in text. Heterogeneity *p* values after Bonferroni correction were *p* < 0.001 for all comparisons, except for Phase I and Phase II (panels D and G), which were not significant at α = 0.05.

Some of the motivation underlying previous eQTL studies has been the observation that eQTLs are enriched for GWAS disease associations^23^ and the GTEx project was motivated to elucidate tissue-level eQTL variation^30^. Here we provide refined findings for liver by devising an enrichment test for various pharmacogenetically important gene sets, expressed as the natural log of odds ratios that the gene has a highly significant *cis*-eQTL (see Methods). For completeness of comparison, we included the UNC Liver and GTEx liver RNA-Seq datasets, as well as results from the microarray liver eQTL meta-analysis^16^. The analyses were not corrected for sample size, as the two RNA-Seq studies were of comparable size, and effect sizes for the microarray datasets had been combined across platforms, and thus are not of meaningful scale. Due to limitations in microarrays for gene expression detection, fewer genes are available from these datasets, and our assumptions for gene set enrichment (see Methods) likely somewhat overstate the enrichment for the microarray meta-analysis. The results are shown in Fig. 2, in terms of odds ratios (natural log scale) for correspondence of highly significant gene-level eQTL findings with gene set membership. The results generally show substantial enrichment of liver eQTLs for all the examined gene sets, with most of the confidence intervals not including zero. The meta-analysis showed greater enrichment than the RNA-Seq studies, which we attribute to its larger sample size, and an upward bias due to fewer genes available for comparison (as discussed in Methods), but the fewer number of detected genes creates limitations for future utility of the dataset. Clear enrichment was present for genes in the NHGRI-EBI catalog of all trait associations (Fig. 2A), as well as for associations with a targeted set of traits considered to be liver-relevant (Fig. 2B, these and other gene sets described here provided in Supplementary File 1). More targeted gene sets included DrugBank Drug Targets (Fig. 2C) and genes involved in various phases of drug metabolism, transport and response (detailed in Methods, Fig. 2D–H). The enrichment for the UNC Liver data vs. GTEx liver was generally comparable, which is consistent with their similar sample sizes, and illustrates the potential power of a resource combining the datasets. Using reasoning similar to that provided in^31^, we also performed the enrichment analyses after a correction for overall gene expression level, as shown in Supplementary Fig. 3. After this correction, the enrichment for all categories was generally present and qualitatively similar, but slightly attenuated, for most of the gene sets examined.

### Splicing association

**Figure 3:**
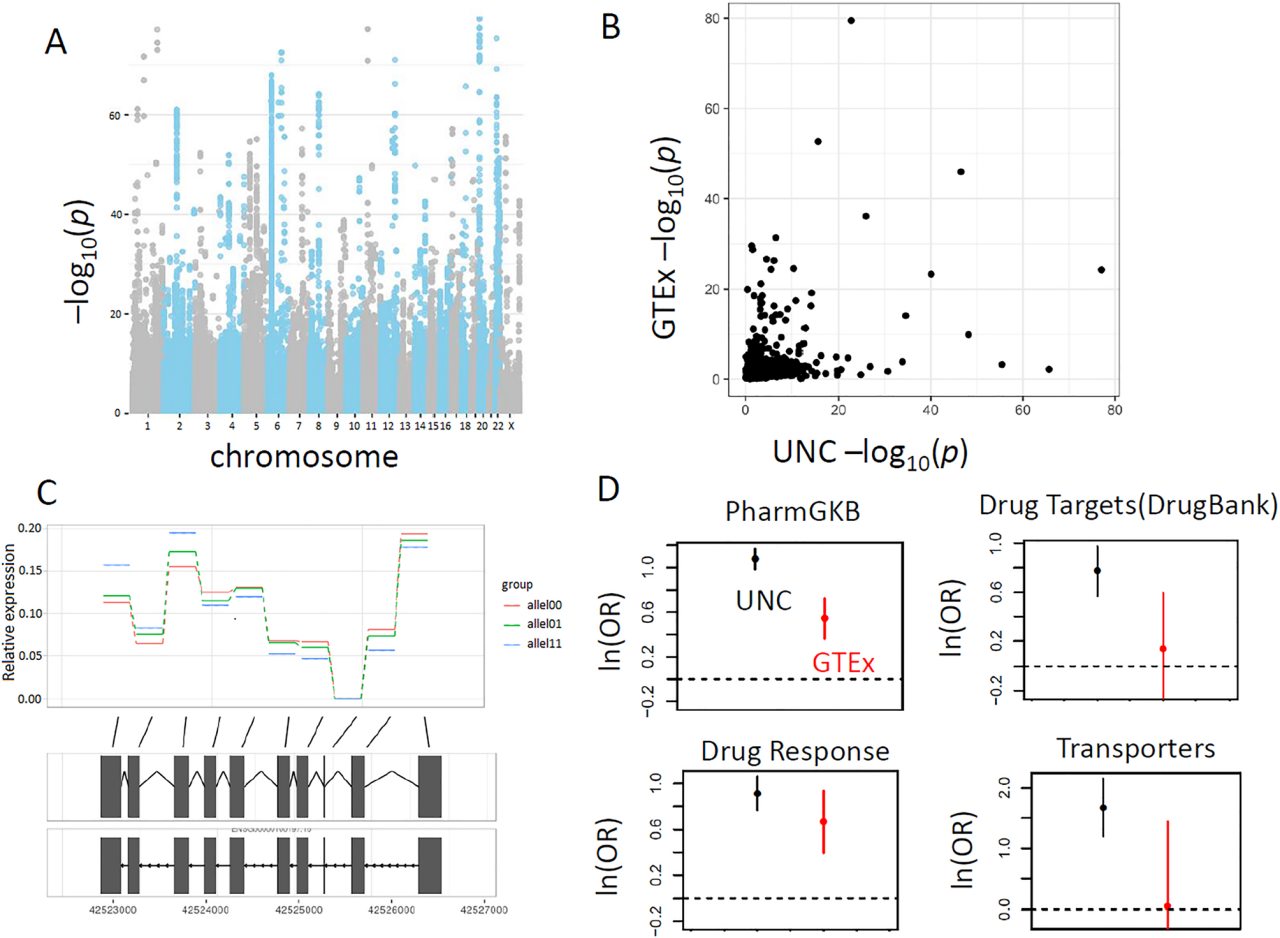
This study is a large compendium of liver splicing regulation information. (**A**) Splicing (exon usage) eQTLs, with minimum *p* value per gene shown. (**B**) Liver splicing eQTL *p* values (minimum per gene) shown, GTEx liver vs. UNC liver (Spearman ρ = 0.19, p < 0.0001). (**C**) Average exon-usage per genotype for rs3892097 shows the effect of the SNP on splice variation on *Cyp2D6*. (**D**) Enrichment of pharmaco-gene sets for gene-level splicing eQTLs. Enrichment for the UNC liver data was significantly greater than that of GTEx liver for PharmGKB (*p* < 0.001 after multiple comparison correction), while other gene categories were suggestive but not significant.

We next performed a splice-variation QTL (sQTL) analysis in the UNC Liver and GTEx liver studies, following the sQTLseekeR approach^28^, which essentially identifies genetic association with exon usage, i.e., proportion of total transcript signal apportioned to each exon. The approach reflects the fact that exon variation underlies isoform variation, which is more difficult to measure directly due to the need to statistically reconstruct full-length transcripts from short sequencing reads^32^. Figure 3A shows a Manhattan plot for the method applied to the UNC Liver data (2192 significant sQTL genes, *q* < 0.1), while Fig. 3B shows that the UNC Liver and GTEx liver data show significant but very modest gene-level consistency in their splicing QTL evidence. The sQTL *p* values are generally less significant than eQTLs, which we attribute the comparatively lower signal, as well as implicit multiple testing required to summarize evidence across exons. Figure 3C shows the exon-usage proportions for *CYP2D6* as a function of genotypes for SNP rs3892097, for illustration. The variant is a splice acceptor site and the minor allele (known as the CYP2D6*4 polymnorphism) results in a non-functional protein (www.snpedia.com/index.php/Rs3892097), and is associated with variation in urinary metabolites^33^ and effects of beta blockers^34^. Figure 3D shows pharmacologically-relevant gene enrichment plots for sQTLs for the UNC Liver and GTEx liver datasets. The enrichment appears generally stronger for the UNC Liver dataset than for GTEx, a difference that is statistically significant (*p* < 0.05) for the PharmGKB, DrugBank, and Transporters gene sets.

### Tissue relationships

Following our focus on liver expression regulation, we reasoned that gene-level (whole mRNA) analysis of tissue-specific eQTL behavior would be instructive. Bayesian modeling of tissue variation in *cis*-eQTLs has been highly instructive^21^, with posterior probabilities for eQTL effects that are highly interpretable. However, fully Bayesian approaches are infeasible for more than ~ 10 tissues^35^, with computational costs that increase exponentially. We utilized the recently developed approximate Bayesian HTeQTL method^29^, which can analyze up to ~ 20 tissues simultaneously and uses the entire collection of tissues to find tissue-tissue correlation structures to increase the power to discover tissue-common eQTLs. For this analysis, we followed the tissue selection of 20 GTEx tissues (primarily based on largest sample size) reported in^29^, to which we added the UNC Liver data as if it were a separate tissue. For each of the 21 tissues/datasets, gene-level summaries were extracted as posterior probabilities that each gene has an eQTL in that tissue (see Methods). These probabilities provide a convenient summary of eQTL evidence, and reflect a full consideration of correlation structures due to both (i) sample overlap, which is a design feature of the GTEx data, and (ii) tissue similarity, reflecting true biological similarities in eQTL effects^35^.

**Figure 4:**
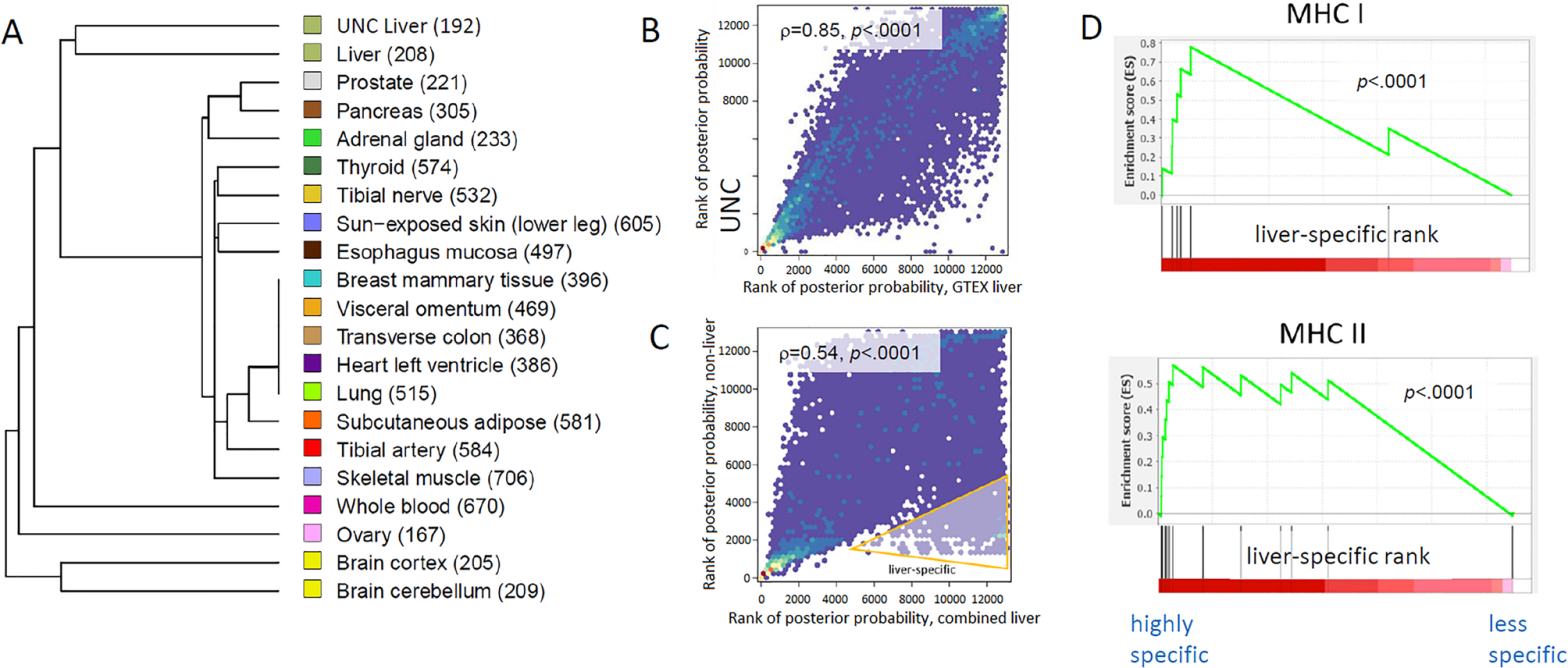
(**A**) Clustering of gene-level eQTL probabilities from the high-tissue Bayesian model shows that the liver studies (UNC and GTEx) are distinct from other GTEx tissues and similar to each other. Color identification scheme is the same as used in^22^. (**B**) UNC liver and GTEx gene-level liver posterior probabilities are highly correlated. (**C**) Comparison of non-liver to combined liver probabilities shows a distinct set of genes that are highly liver-specific for eQTLs. (**D**) GSEA plots: HLA/MHC genes are highly enriched among liver-specific eQTL genes, especially for MHC Class II genes.

Figure 4A shows the results of hierarchical clustering of the tissues based on gene-level eQTL probabilities, which reflect the tissue eQTL similarity rather than mere gene–gene correlation between tissues. Several features are notable. Tissues such as brain, ovary, and whole blood appear quite distinct from other tissues. The two liver datasets also appear quite distinct from other tissues, and most similar to each other, providing reassurance about reproducibility of liver eQTL effects. Figure 4B shows the posterior probabilities across the 28,047 genes for GTEX v8 liver vs. UNC Liver, shown on the rank scale because a large number of genes have been shown to be associated with eQTLs with high probabilities^21^. We further reasoned that liver-specific eQTLs would be of special interest. The HT-eQTL software can provide, for each gene, an estimated posterior probability that the gene is influenced by a *cis*-eQTL. Accordingly, we plotted the rank of these posterior probabilities for liver eQTLs (combining GTEx and UNC Liver by averaging) vs. the averages for the non-liver tissues (Fig. 4C). The figure conceptually highlights a region containing genes with high rank for liver eQTL effects, but low rank for non-liver eQTL effects. The nature of the HT-eQTL model does not enable a fully probabilistic inference for tissue-specific expression across two datasets, so we devised a simple measure of liver specificity as a ratio of eQTL posterior probabilities within vs. across tissues (see Methods). The utility of such a measure is primarily in providing gene rankings, and we performed gene set enrichment analysis of the ranked genes (Table 1). A clear enrichment of HLA/MHC genes was apparent (*p* < 0.0001) and inspection of the individual highly-ranked genes suggested a potential difference between MCH Class I and Class II genes. We created a custom categorization based on Class I/II genes, and Fig. 4D shows the enrichment results, expressed as a GSEA plot^36^.

**Table 1:** Pathway enrichment of liver specificity of genes with cis-eQTLs.

DAVID/EASE			
Cluster	Data source	P value	Q value
MHC classes I/II	InterPro, SMART, UniProt, GO CC, GO BP, GO MF, KEGG	8.7 × 10^–9^	7.6 × 10^–6^
Mitochondrion	UniProt, GO CC	2.1 × 10^–6^	2.4 × 10^–4^
Krueppel-associated box (KRAB)	SMART	4.3 × 10^–5^	2.8 × 10^–3^
Ribosome	UniProt, KEGG	4.5 × 10^–4^	1.9 × 10^–2^
DNA repair	UniProt	1.6 × 10^–3^	5.3 × 10^–2^

IPA			
Pathway		P value	Q value
Antigen presentation pathway		1.8 × 10^–7^	5.5 × 10^–5^
Allograft rejection signaling		1.5 × 10^–6^	2.3 × 10^–4^
OX40 signaling pathway		5.0 × 10^–6^	5.0 × 10^–4^
Phagosome maturation		3.0 × 10^–5^	2.2 × 10^–3^
Autoimmune thyroid disease signaling		5.1 × 10^–5^	2.8 × 10^–3^
Cdc42 signaling		5.5 × 10^–5^	2.8 × 10^–3^
Graft-versus-host disease signaling		6.9 × 10^–5^	3.0 × 10^–3^
Autophagy		2.3 × 10^–4^	8.7 × 10^–3^
B cell development		5.2 × 10^–4^	1.8 × 10^–2^
PD-1, PD-L1 cancer immunotherapy pathway		7.4 × 10^–4^	2.2 × 10^–2^
Acyl-CoA hydrolysis		2.5 × 10^–3^	6.5 × 10^–2^
Glutathione-mediated detoxification		2.6 × 10^–3^	6.5 × 10^–2^
Th2 pathway		4.3 × 10^–3^	1.0 × 10^–1^

### An illustration of transcriptome-wide association

**Figure 5:**
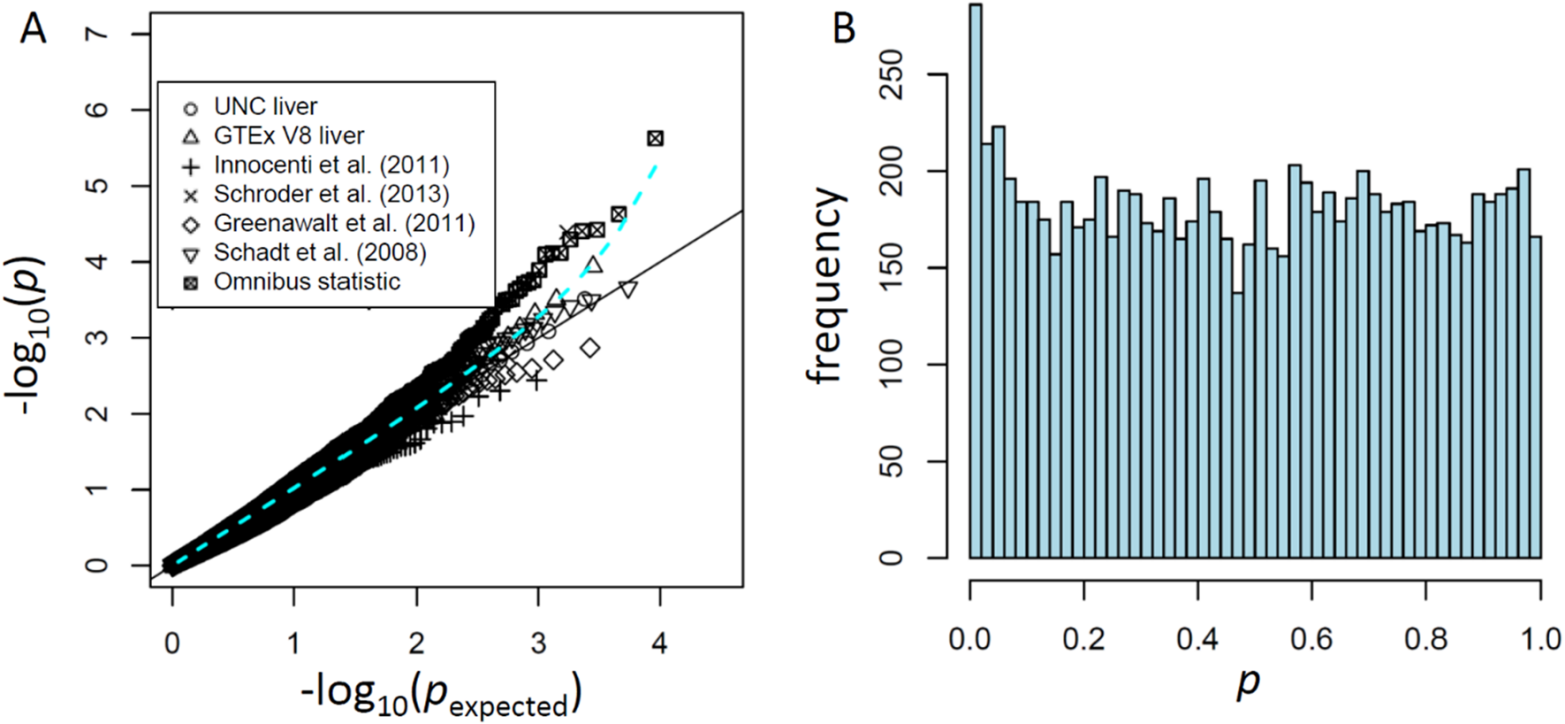
A small neutropenia GWAS study (*n* = 294) illustrates the power of TWAS analysis from combined liver eQTL sources. (**A**) Quantile–quantile plot of − log10(*p* values) for TWAS analyses using four earlier liver eQTL studies and two RNA-Seq liver eQTL studies. (**B**) Histogram of *p* values for the omnibus statistic.

One important use of eQTL studies is in the calculation of transcriptome-wide association (TWAS) *p* values for genetic association^37^, which uses expression imputation to provide strong evidence for a directional expression-based hypothesis for association^23,37^. We use as a proof of principle the study of treatment-induced neutropenia^38^ in *n* = 294 pancreatic cancer patients treated with gemcitabine, using the six liver eQTL studies (Supplementary Table 1) as reference dataset input. The omnibus statistic proposed by^37^ is intended to combine information across multiple eQTL reference datasets, but for these data appeared to potentially produce false positive results, based on permutation investigations. Upon further analysis, we observed that the omnibus method^37^ appeared to be highly sensitive to the correlation of the eQTL reference datasets, in a manner that is not fully controlled in the original method. We thus devised an approach (Methods) to account for this excess correlation, providing more accurate omnibus *p* values and harnessing the increased power due to consistent direction of expression association. In addition, our proposed approach can easily account for missing information, which occurs when gene expression is available in only some of the datasets. The results for our statistics are shown in Fig. 5A, with several genes reaching transcriptome-wide significance. The histogram of *p* values (Fig. 5B) illustrates that the overall distribution is reasonably uniform, but with a spike near zero, as expected if the false-positive rate has been controlled. Genes with false-discovery TWAS *q* < 0.15 were *UGT1A3*, *SLC6A13*, *EIF3I*, *UGT1A2P*, and *CLDN3*. The TWAS analysis also identified increased *MFAP5* expression at suggestive significance (*p* < 0.0003, *q* = 0.17) associated with decreased risk of neutropenia. In addition to the individually significant genes, a DAVID/EASE pathway analysis for “high-density lipoprotein (HDL) particle” (CC) gave false discovery *q* = 0.037.

## Discussion

We have intended this dataset to be used as a comparative resource, and for examining the effects of individual genes across a combination of liver datasets. Several of the top-line findings reinforce the utility of the dataset and relevance of the identified genes. For example, the UNC Liver analysis identified endoplasmic reticulum aminopeptidase 2 (*ERAP2*) as the most significant liver eGene. GTEx also identified *ERAP2* as a highly significant eGene (*q* = 3.6 × 10^–61^), with only one eGene with a lower *q value*. *ERAP2* functions in the immune antigen-processing pathway by trimming basic amino acid residues at the N-terminus of polypeptides to generate optimal peptide lengths for loading onto MHC molecules, thus shaping the repertoire of antigens presented to T cells both qualitatively and quantitatively^39^. The human liver is comprised of approximately 60–70% hepatocytes by mass, with the remaining 30–40% consisting of a diverse spectrum of innate and adaptive immune cells^40^. Polymorphisms in *ERAP2* have been associated with predisposition to a number of autoimmune^41–43^ and infectious diseases, including chronic hepatitis C infection^44–47^. These polymorphisms are in high LD (*r*^2^ > 0.75) with the leading eQTL for *ERAP2* identified in this study, rs2910788, which was associated with increased *ERAP2* expression. Variants that increase the gene expression of *ERAP2*, including rs2910788, have been consistently associated with increased disease risk (Ye et al. 2010; PMID: 29108111). *ERAP2* might also have a central role in immune-mediated adverse reactions by interacting with the HLA system^48^. So far, the only observation reported has been the interaction of genetic variation in *ERAP2*, in high LD with rs2910788 reported here, and HLA-C*04:01 influencing the risk of Stevens-Johnson syndrome/toxic epidermal necrosis secondary to nevirapine^49^. Our study points to the need for an evaluation of *ERAP2* for immune-related adverse effects of medications.

The most statistically significant *trans*-eQTL in our study was rs10413980 (C > T), which was associated (*p* = 2.7 × 10^–16^) with the expression of flavin containing dimethylaniline monooxygenase 2 (*FMO2*). The rs10413980 variant is also a *cis*-eQTL of zinc finger protein 160 (*ZNF160*). ZNF160 belongs to the largest individual class of transcriptional repressors^50–52^. The T allele of rs10413980 was associated with increased *ZNF160* expression and decreased *FMO2* expression, suggesting the possibility that rs10413980 regulates *FMO2* expression through the repressor activity of *ZNF160*. *FMO2* is the predominant *FMO* isoform present in lung^53^ and catalyzes the metabolism of therapeutic drugs including ethionamide which is used to treat multidrug-resistant tuberculosis^54–56^. GTEx did not identify any *trans*-associations in liver and did not identify rs10413980 as a lung *trans*-eQTL. It is noteworthy that *FMO2* expression has a strong genetic *cis-*regulation, due to the FMO2*2 variant encoding a truncated, nonfunctional protein^57,58^. *FMO2*-mediated metabolism may affect both the efficacy and toxicity of ethionamide^59^. Therefore, in populations expressing the full length *FMO2,* differences in *FMO2* expression resulting from *trans*-eQTL regulation by rs10413980 may explain some of the interindividual variation in ethionamide pharmacokinetics and potential impacts on therapeutic efficacy and toxicity (PMID: 12356460). Here we suggest a novel mechanism for the regulation of *FMO2* expression in the human liver.

The two findings of *ERAP2* and the liver-specific enrichment of eQTLs in MHC genes point the attention to the issue of liver tissue heterogeneity and the biological roles of liver-related immune cells. Hepatocytes are capable of presenting antigens using the MHC class I pathway. However, only a few cell types (i.e. dendritic, Kupffer), considered professional antigen presenting cells, are able to prime naïve T cells. Liver-resident lymphocytes, with phenotypes distinct from those in circulation^60^, potentially play a key role in a variety of processes in response to infection and injury^61,62^. However, considering that hepatocytes are the predominant liver cell type^63–65^ and we use bulk RNA-Seq, this study cannot discern the cell-type origin of the reported signals. For GTEx liver, very few hepatocyte cell-type eQTLs were discovered^66^. Single-cell sequencing studies^67^ are still in early phases, and computational methods to deconvolute cell type-specific eQTLs vary in power^68^, with very few significant findings reported for liver hepatocytes^66^. Thus, current eQTL studies using bulk RNA remain predominant, and first-line analyses^22^ approach cell-type variation largely in terms of covariate control, especially in order to avoid false positive findings for loci that affect cell type proportions within tissue^31^.

Given the cellular composition of liver, the highly polymorphic nature of the MHC^69^, the critical role of the liver as a buffer between gut contents and the systemic circulation, and the susceptibility of the liver to viral infection, enrichment of eQTL in immune-related genes representing both MHC class I and II is consistent with the immunological composition and function of the liver and differences in gene expression may help to explain individual differences in susceptibilities to viral infection and other liver related diseases.

Our TWAS neutropenia example further illustrates the utility of a liver eQTL resource. For the suggestive TWAS association of *MFAP5*, we note that^70^ have demonstrated that the Mfap5 knockout in mouse models results in decreased levels of circulating neutrophils and that MAGP2, the protein product of *MFAP5*, binds to members of the TGFβ superfamily. TGFβs are master regulators of hematopoietic stem cell quiescence suggesting regulation of TGFBs by increased expression of MGAP2 may result in increased hematopoiesis.

In conclusion, we describe here a liver eQTL resource including results from linear expression association, allelic fold change, and exon usage-based splicing eQTL analyses. We demonstrate the utility of this resource for providing mechanistic insights into genetic associations through the example of variation in (1) *ERAP2* and predisposition to autoimmune and infectious diseases, (2) *FMO2* and interindividual variation in ethionamide pharmacokinetics and potential efficacy and toxicity, and (3) *MFAP5* neutropenia induced by chemotherapy. We make this resource available at https://seeqtl.org/.

## Methods

### Sample QC

GWAS genotype data from the Illumina Human610-Quad v1.0 BeadChip and RNA-Seq expression data derived from liver tissues (described in Etheridge et al. 2020) using the Illumina HiSeq2000 was available from 206 individuals. Four samples with a genotype call rate < 98% across samples were excluded. Genotype data was used to calculate pairwise identity by descent (IBD) in PLINK to check for replicated samples or first-degree relatives. PLINK was also used to calculate heterozygosity rates from genotype data on X chromosome SNPs to determine the sex of the individual providing the liver sample. Expression data from the X-linked gene *XIST* and the Y-linked gene *RPS4Y1* was used to identify males and females. Four samples whose sex did not match between reported sex and the sex determined from both genotyping and expression were also excluded. Expression data was also used to check for errors in sample handling, including samples that might have been mislabeled or swapped. The top 100 genes associated with the most significant *cis*-eQTLs (in terms of Pearson *r*, genotype vs. expression) were identified and 100 linear models of expression ~ genotype were fitted. These models and genotypes from each sample were then used to predict expression levels for samples. Correlations between actual and predicted expression levels were determined and the maximal correlation across samples, corresponding to the actual expression data with the highest correlation to predicted expression, were identified. In the case of proper sample handling, maximal correlations are expected between each sample and itself. In instances where the maximal correlation of a sample corresponded to a sample other than itself, both samples were excluded from analysis, following similar reasoning as^71^. Based on the criteria described above, six samples were excluded, and the number of samples remaining was *n* = 192 (124 males and 68 females).

### RNA-Seq pre-processing

Reads were trimmed of adapter and for quality using Trimmomatic v0.35 leaving paired reads with a minimum length of 20 bp. Trimmed reads were mapped to the human genome reference hg19/GRCh37 via HiSat2. Alignment files were sorted and converted into bam files and Picard Tools used to mark duplicates in the alignments. Resulting bam files were indexed. Counts for exons, genes and TPMs (Transcripts per Kilobase Million) were generated with RNASeqC v2.1 in “legacy” mode with the Gencode.v19.gene.v7.patched_contigs.gtf annotation file as a guide. Count files were then aggregated into single files for each of exon reads, gene reads and gene TPMs with a Python script. The 29,245 gene transcripts with expression thresholds of > 0.1 TPM and ≥ 6 reads in at least ten individuals were selected for analysis. The distribution of TPMs in each sample was quantile normalized to the average empirical distribution observed across all samples. The normalized TPMs for each gene were subsequently transformed to the quantiles of the standard normal distribution.

### Genotype pre-processing and imputation

Genotype data was imported into PLINK for quality control filtering. Variants with a call rate below 95% across samples, a minor allele frequency (MAF) < 0.01, or with significant deviation from Hardy–Weinberg equilibrium (HWE, *p* value < 1 × 10^–6^) were excluded prior to imputation. Stranding was verified for all remaining variants to ensure alignment with the 1000 Genomes Project reference population. In instances where a stranding mismatch was identified, alleles were either flipped to match those in the 1000 Genomes Project data or variants were excluded due to ambiguous stranding. In order to supplement the directly interrogated genotypes on the platform, genotype imputation was performed for each chromosome. A two-step process was performed for imputation. First, QC-filtered genotype data was utilized for sample haplotype estimation using the MACH (http://csg.sph.umich.edu/abecasis/MACH/index.html) software package. Phased haplotypes were subsequently used in Minimac2 (http://genome.sph.umich.edu/wiki/Minimac2) to impute missing genotypes using the 1000 Genomes Project Phase 1 haplotypes as a reference. The information metric from Minimac2 was used to determine variants that were imputed with a high degree of certainty and variants with a *r*^2^ quality score < 0.3 were excluded from analysis. In addition, variants that had a MAF < 0.01 were excluded from analysis. Dosages in terms of the minor allele were calculated for the 10,856,510 variants remaining in the analysis.

### Covariates

Sex, age, genetic ancestry, and experimental batch effects were included as covariates in the analysis. The covariates for genetic ancestry were generated by running a principal component analysis of the genotype data using smartpca in the EIGENSOFT software package^72^. The first three principal components were determined to be significant. We applied correction of potential confounders in the expression data by controlling for 30 probabilistic estimation of expression residuals (PEER) factors, following^19^.

### eQTL mapping

*cis*-eQTL mapping was performed using the ACMEeQTL (https://github.com/andreyshabalin/ACMEeqtl)^26^ and Matrix-eQTL^24^(http://www.bios.unc.edu/research/genomic_software/Matrix_eQTL) software packages. Matrix-eQTL uses a linear model, and expression values were rank inverse transformed to follow a normal distribution prior to analysis. ACMEeQTL uses a log of linear model (i.e. both expression and the linear term are subjected to natural log transformation) applied to the TPM values. The mapping window was defined as 1 Mb to either side of the transcription start site (TSS) to identify *cis*-variants. Minor allele dosage of variants was used along with covariates to examine the association with processed expression data. The resulting *t*-statistics for each transcript-variant pair were used to generate *p* values. An “eGene” analysis based on the FastQTL software^27^ was used to generate gene-level *p* values from the most significant *cis*-SNP. The significance of the most highly associated variant per gene was determined from empirical *p* values (10^6^ permutations), extrapolated from a beta distribution fitted to adaptive permutations. Results of these *cis*-eQTL analyses were integrated with liver eQTL data on 208 individuals in release V8 of the Genotype-Tissue Expression (GTEx) Project. *Trans*-eQTL mapping was performed using Matrix-eQTL to identify *trans*-variants that were on a different chromosome from the target gene. The regionally most significant eQTL was determined for putative *trans*-eQTL associations with FDR *q* < 0.1, and then subjected to additional quality control for (i) cross-mappability with another gene, following the criteria used in^22^, (ii) more than 2 significant eQTL regions associated with a single gene, (iii) a poorly annotated target gene with no gene symbol, (iv) a target gene that is a pseudogene. Only *trans*-associations with none of these potential flags was considered “high-quality,” an approach that leaves the possibility of false negatives. False discovery rate (FDR) control was applied for each set of findings using the Benjamini–Hochberg approach, with *cis*- and *trans*- tests corrected separately.

### Splicing-QTLs

The sQTLseekeR software package (https://github.com/guigolab/sQTLseekeR)^28^ was used to detect splicing QTLs (sQTLs), which are variants associated with change in the splicing pattern of a gene. Here, splicing patterns are represented by the relative expression of exons in a gene as a proportion of all mapped reads. This software can assess the association between the genotype at a given variant and the splicing ratios of a given gene, using a multivariate approach, providing resampling-based gene-level *p* values (10^8^ permutations) that were then subjected to Benjamini–Hochberg FDR control.

### Designation for genes involved in drug response

The NHGRI-EBI catalog (www.ebi.ac.uk/gwas/) was accessed and downloaded in March, 2020, and genes were cataloged as having associations in the catalog or not. A list of 1496 genes of drug response was compiled from the Pharmacogenomics Knowledge Base (PharmGKB) (http://www.pharmgkb.org/) (January, 2017), a comprehensive database that curates information about the impact of genetic variation on drug response; the PharmaADME Working group list of absorption, distribution, metabolism, and excretion genes (http://www.pharmaadme.org); the US Food and Drug Administration (FDA) Pharmacogenomics Biomarkers (https://www.fda.gov/drugs/science-and-research-drugs/table-pharmacogenomic-biomarkers-drug-labeling), the Nuclear Receptor Signaling Atlas (NURSA) Consortium^73^; the DrugBank catalog (https://www.drugbank.ca/), a comprehensive database containing information on drug targets; and the literature^74,75^. This list was used to filter eQTLs for association with genes of drug response. The drug target gene set was compiled from the DrugBank catalog and is a list of genes that are targeted by approved drugs. Drug metabolism enzymes were divided into Phase I and Phase II, and “Transporters” used to denote liver transport enzymes. The PharmGKB gene set^76^ was compiled from the PharmGKB database and included all curated variants with gene–drug relationships regardless of level of evidence. Supplementary File 1 shows the full set of indicators (Yes/No) whether each gene belongs to each of the gene sets.

### Enrichment for liver-relevant gene sets

To provide an easily interpretable enrichment statistic, for liver-relevant pharmacogenetically important gene sets, we used logistic regression to provide natural log odds ratios at the gene level for gene set membership and the presence/absence of a nominally significant *cis*-eQTL or eGene (*p* < 10^–5^). The UNC Liver and GTEx Liver datasets are of approximately the same size (~ 200 samples), and thus this simple approach can provide an effective comparison. We used the same approach for the microarray meta-analysis, although with a larger sample size the power (and therefore log odd ratios) was expected to be considerably larger. However, the expression detection limitations for microarrays provide a challenge. Using only the genes considered to be above detection limits produced little evidence of gene set enrichment. We opted to consider all genes, by assuming genes with lower expression as “not significant,” which likely exaggerated the log odds ratios for the microarray meta-analysis, because higher expression genes are more likely to have significant eQTLs and to belong to well-studied pathways. For the UNC Liver and GTEx Liver datasets we also produced a log odds ratio for enrichment, after correcting for the TPM average expression level for each gene in the logistic regression model. Tests of heterogeneity of enrichment reflect differences in the log odds ratios, and were tested using Cochran’s Q. For the sQTLSeeker results, we performed the analogous analyses in the same manner.

### Multi-tissue analysis and tissue specificity

The HT-eQTL method (https://github.com/reagan0323/MT-eQTL/tree/master/HT-eQTL)^29^ was applied to the UNC Liver dataset along with 19 additional GTEx V8 datasets, including two brain tissues, two adipose tissues, and a heterogeneous set of 16 other tissues including liver. This method jointly analyzes all *cis* pairs to enable the characterization of tissue specificity. The results of this analysis are probabilities that for a given *cis* pair, an eQTL is detected across any tissue, across all tissues, or specifically in one tissue. The methods of^29^ were used to provide a profile of probabilities, for each gene, whether the gene has a *cis*-eQTL in a particular tissue. These probability profiles (of length 21) were then used as vectors for hierarchical clustering of tissues, using a distance matrix of 1-ρ (Spearman correlation) and average linkage.

The Bayesian analysis revealed that a large proportion of genes have *cis*-eQTL associations in a large variety of tissues, even if weak^19^. The concept of tissue-specificity is then largely a matter of degree. Additionally, computing formal posterior probabilities for the combined liver datasets was not possible from the individual datasets, as several summation steps by HT-eQTL are done within each tissue (set of samples). Thus, we adopted a rank-based procedure that was less formal but served to point to potential pathway findings. For each of the UNC Liver and GTEx datasets and the set of all genes, the rank was computed for the posterior probability that the gene had a *cis*-eQTL. The ranked vectors for liver and non-liver were contrasted by computing scores for each gene, score1 = (rank of UNC Liver + rank of GTEx liver) and score2 = (mean rank in all non-liver tissues). Then an approximate liver-specificity score was computed as specificity = score1/score2. The top 200 genes for this liver-specificity score were then used for DAVID/EASE (https://david.ncifcrf.gov/, version 6.8) and Ingenuity pathway analysis, while the liver-specificity score for all genes were used in the investigation of MHC pathway enrichment using GSEA^36^ (https://www.gsea-msigdb.org).

### TWAS analysis

A transcriptome-wide association study (TWAS) analysis (http://gusevlab.org/projects/fusion)^37^ was performed to identify associations between a GWAS phenotype and a functional phenotype that was only measured in reference data. First gene expression weights from the UNC Liver and the 20 GTEx datasets, and four microarray datasets (“tissue”), were computed as the reference datasets. GWAS summary statistics for the neutropenia-related phenotype were available, and by following^37^ an association *z*-statistic vector ${z}_{i}$ for the neutropenia phenotype across all genes was obtained for each tissue *i*. Separate association results were generated for each reference dataset as z-statistics. The omnibus statistic in^37^ that is based on association *p* values was investigated using null permutations of the neutropenia-related phenotype from^38^, which should have resulted in nearly uniform *p* values. However, numerous false positives appeared, even under multiple test corrections, which we attributed to high correlations among the GTEx samples. We thus devised an alternative directional omnibus statistic, $z=\sum_{i}{z}_{i}/\sqrt{v}$, where $v=\sum_{i}\sum_{j}{\rho }_{ij}$ and ${\rho }_{ij}$ is the observed correlation across the genes between ${z}_{i}$ and ${z}_{j}$. As a final measure to ensure robustness, an approach similar to^77^ was used to linearly re-scale the resulting omibus *z*-statistics so that the 0.25 and 0.75 quantiles of the resulting distribution matched the corresponding quantiles of a standard normal.

## Supplementary Information

Supplementary Information 1.

Supplementary Table 1.

Supplementary Table 2.

Supplementary Figure 1.

Supplementary Figure 2.

Supplementary Figure 3.

## Data Availability

Genotype data for the UNC Liver study are made available as GEO submission GSE 26105, and expression data will be made available in July 2022, GSE 179250.
