# Supplementary material for: A resource for integrated genomic analysis of the human liver
Source: Sci Rep. 2022 Sep 7;12:15151. doi: 10.1038/s41598-022-18506-z (PMC9452507; doi:10.1038/s41598-022-18506-z)
Supplement: Supplementary file 2 — Supplementary Table 1. [file 41598_2022_18506_MOESM2_ESM.docx]

**Supplementary Table 1**

| **Data source** | **Sample size** | **Tissue** | **Expression platform** | **Genotype platform** | **Number of genes used/ expressed** | **Number of SNPs used** |
| --- | --- | --- | --- | --- | --- | --- |
| UNC RNA-Seq | 192 | Liver | RNA-Seq | Illumina Human610-Quad v1.0 | 29,245 | 10,856,510 |
| [GTEx v8](https://www.biorxiv.org/content/10.1101/787903v1) | 208 | Liver | RNA-Seq | Illumina HiSeq | 22,262 | 9,823,615 |
| [Etheridge et al.](https://www.ncbi.nlm.nih.gov/pmc/articles/PMC7816646/) | 1,183 | Liver |  |  |  |  |
| [Schroder et al.](https://www.ncbi.nlm.nih.gov/pmc/articles/PMC3564008/) | 145 | Liver | Illumina Human Whole Genome-6 v2.0 | Illumina HumanHap300-Duo v2.0 | 42,540 | 7,887,016 |
| [Innocenti et al.](https://www.ncbi.nlm.nih.gov/pmc/articles/PMC3102751/) | 161 | Liver | Agilent-014850 Whole Human Genome 4x44K | Illumina Human610-Quad v1.0 | 30,063 | 8,282,977 |
| [Schadt et al.](https://www.ncbi.nlm.nih.gov/pmc/articles/PMC2365981/) | 322 | Liver | Agilent Technologies (Custom ~40K transcripts) | Affymetrix GeneChip Human Mapping 500K | 35,195 | 6,748,335 |
| [Greenawalt et al.](https://www.ncbi.nlm.nih.gov/pmc/articles/PMC3129244/) | 555 | Liver | Agilent Technologies (Custom ~40K transcripts) | HumanHap 650Y | 33,911 | 7,979,715 |
|  |  |  |  |  |  |  |
| [GTEx v8](https://www.biorxiv.org/content/10.1101/787903v1) | 581 | Adipose Subcutaneous | RNA-Seq | Illumina HiSeq | 24,665 | 10,531,780 |
| [GTEx v8](https://www.biorxiv.org/content/10.1101/787903v1) | 469 | Adipose Visceral Omentum | RNA-Seq | Illumina HiSeq | 24,724 | 10,298,421 |
| [GTEx v8](https://www.biorxiv.org/content/10.1101/787903v1) | 233 | Adrenal Gland | RNA-Seq | Illumina HiSeq | 23,820 | 10,192,919 |
| [GTEx v8](https://www.biorxiv.org/content/10.1101/787903v1) | 584 | Artery Tibial | RNA-Seq | Illumina HiSeq | 23,304 | 10,595,553 |
| [GTEx v8](https://www.biorxiv.org/content/10.1101/787903v1) | 209 | Brain Cerebellum | RNA-Seq | Illumina HiSeq | 25,461 | 9,779,227 |
| [GTEx v8](https://www.biorxiv.org/content/10.1101/787903v1) | 205 | Brain Cortex | RNA-Seq | Illumina HiSeq | 24,849 | 9,798,483 |
| [GTEx v8](https://www.biorxiv.org/content/10.1101/787903v1) | 396 | Breast Mammary Tissue | RNA-Seq | Illumina HiSeq | 25,849 | 10,445,218 |
| [GTEx v8](https://www.biorxiv.org/content/10.1101/787903v1) | 368 | Colon Transverse | RNA-Seq | Illumina HiSeq | 25,379 | 10,424,038 |
| [GTEx v8](https://www.biorxiv.org/content/10.1101/787903v1) | 497 | Esophagus Mucosa | RNA-Seq | Illumina HiSeq | 23,949 | 10,515,285 |
| [GTEx v8](https://www.biorxiv.org/content/10.1101/787903v1) | 386 | Heart Left Ventricle | RNA-Seq | Illumina HiSeq | 21,353 | 10,316,107 |
| [GTEx v8](https://www.biorxiv.org/content/10.1101/787903v1) | 515 | Lung | RNA-Seq | Illumina HiSeq | 26,095 | 10,336,822 |
| [GTEx v8](https://www.biorxiv.org/content/10.1101/787903v1) | 706 | Muscle Skeletal | RNA-Seq | Illumina HiSeq | 21,031 | 10,524,672 |
| [GTEx v8](https://www.biorxiv.org/content/10.1101/787903v1) | 532 | Nerve Tibial | RNA-Seq | Illumina HiSeq | 25,873 | 10,525,826 |
| [GTEx v8](https://www.biorxiv.org/content/10.1101/787903v1) | 167 | Ovary | RNA-Seq | Illumina HiSeq | 25,325 | 10,044,857 |
| [GTEx v8](https://www.biorxiv.org/content/10.1101/787903v1) | 305 | Pancreas | RNA-Seq | Illumina HiSeq | 22,615 | 10,264,916 |
| [GTEx v8](https://www.biorxiv.org/content/10.1101/787903v1) | 221 | Prostate | RNA-Seq | Illumina HiSeq | 26,529 | 10,227,455 |
| [GTEx v8](https://www.biorxiv.org/content/10.1101/787903v1) | 605 | Skin Sun Exposed Lower Leg | RNA-Seq | Illumina HiSeq | 25,196 | 10,455,579 |
| [GTEx v8](https://www.biorxiv.org/content/10.1101/787903v1) | 574 | Thyroid | RNA-Seq | Illumina HiSeq | 26,054 | 10,355,053 |
| [GTEx v8](https://www.biorxiv.org/content/10.1101/787903v1) | 670 | Whole Blood | RNA-Seq | Illumina HiSeq | 20,315 | 10,526,303 |
