# Supplementary material for: A resource for integrated genomic analysis of the human liver
Source: Sci Rep. 2022 Sep 7;12:15151. doi: 10.1038/s41598-022-18506-z (PMC9452507; doi:10.1038/s41598-022-18506-z)
Supplement: Supplementary file 3 — Supplementary Table 2. [file 41598_2022_18506_MOESM3_ESM.docx]

**Supplementary Table 2.** Significant trans-eQTL results from UNC and GTEx v8 liver

**Ensembl ID^a^ Gene symbol Gene chr. Gene TSS eQTL^b^ eQTL z_UNC_^c^ *p*_UNC_ z_GTEx_ *p*_GTEx_  z_meta_ *p*_meta_ min *p* crossmap quality^e^**

**chr bp score^d^**

| ENSG00000094963.9 | FMO2 | 1 | 171154347 | 19 | 53595330 | -8.2 | 2.71E-16 | NA | NA | NA | NA | 2.71E-16 | 0.969 | HQ |
| --- | --- | --- | --- | --- | --- | --- | --- | --- | --- | --- | --- | --- | --- | --- |
| ENSG00000100883.7 | SRP54 | 14 | 35451163 | 11 | 45201076 | -7.7 | 1.40E-14 | NA | NA | NA | NA | 1.40E-14 | 1.000 | HQ |
| ENSG00000100883.7 | SRP54 | 14 | 35451163 | 13 | 1.07E+08 | -7.6 | 2.66E-14 | NA | NA | NA | NA | 2.66E-14 | 1.000 | HQ |
| ENSG00000165480.11 | SKA3 | 13 | 21750741 | 11 | 1.09E+08 | 7.6 | 3.61E-14 | NA | NA | NA | NA | 3.61E-14 | 1.000 | HQ |
| ENSG00000171365.11 | CLCN5 | 23 | 49687225 | 17 | 57142253 | -7.4 | 1.38E-13 | NA | NA | NA | NA | 1.38E-13 | 0.981 | HQ |
| ENSG00000215030.4 | RPL13P12 | 17 | 17287326 | 16 | 89623990 | 12.4 | 2.49E-35 | 16.4 | 8.84E-61 | 20.4 | 1.65E-92 | 1.65E-92 | 0.744 | CM |
| ENSG00000227097.4 | RP11-742N3.1 | 11 | 82400571 | 19 | 8387207 | -19.4 | 5.67E-84 | -8.9 | 5.38E-19 | -20.0 | 3.32E-89 | 3.32E-89 | 0.341 | CM |
| ENSG00000253570.1 | RNF5P1 | 8 | 38458718 | 6 | 32147696 | 11.7 | 1.73E-31 | 14.8 | 1.51E-49 | 18.7 | 3.50E-78 | 3.50E-78 | 0.804 | CM |
| ENSG00000240342.2 | RPS2P5 | 12 | 118683877 | 16 | 2011126 | -10.8 | 3.49E-27 | -15.6 | 1.22E-54 | -18.6 | 1.44E-77 | 1.44E-77 | 0.156 | CM |
| ENSG00000253626.2 | EIF5AL1 | 10 | 81272357 | 17 | 7215142 | 12.4 | 1.86E-35 | 13.7 | 1.65E-42 | 18.4 | 5.23E-76 | 5.23E-76 | 0.472 | CM |
| ENSG00000197149.4 | RP11-452G18.2 | 11 | 17249817 | 16 | 2014031 | -13.3 | 1.60E-40 | -12.4 | 1.45E-35 | -18.2 | 3.22E-74 | 3.22E-74 | 0.473 | CM |
| ENSG00000213025.2 | COX20P1 | 10 | 70392128 | 1 | 2.45E+08 | 11.2 | 2.81E-29 | 13.9 | 3.59E-44 | 17.8 | 7.02E-71 | 7.02E-71 | 0.772 | CM |
| ENSG00000237039.1 | AC018738.2 | 2 | 232120810 | 19 | 8395410 | 13.0 | 6.57E-39 | 11.5 | 7.60E-31 | 17.4 | 9.60E-68 | 9.60E-68 | 0.286 | CM |
| ENSG00000214110.3 | LDHAP4 | 9 | 14922332 | 11 | 18428264 | -11.8 | 2.40E-32 | -12.1 | 9.78E-34 | -16.9 | 2.55E-64 | 2.55E-64 | 0.829 | CM |
| ENSG00000214455.3 | RCN1P2 | 13 | 45965166 | 11 | 32123016 | -13.6 | 4.88E-42 | -10.2 | 1.19E-24 | -16.9 | 9.84E-64 | 9.84E-64 | 0.704 | CM |
| ENSG00000229644.4 | NAMPTL | 10 | 36813162 | 7 | 1.06E+08 | -10.5 | 1.14E-25 | -12.7 | 8.30E-37 | -16.4 | 3.27E-60 | 3.27E-60 | 0.587 | CM |
| ENSG00000231245.2 | C1DP1 | 10 | 32800436 | 2 | 68281759 | 11.3 | 1.96E-29 | 11.7 | 1.81E-31 | 16.2 | 3.81E-59 | 3.81E-59 | 0.465 | CM |
| ENSG00000224114.1 | RP11-343H5.4 | 1 | 206869614 | 5 | 1.5E+08 | 8.7 | 3.45E-18 | 14.0 | 1.05E-44 | 16.1 | 4.28E-58 | 4.28E-58 | 0.864 | CM |
| ENSG00000266066.1 | POLRMTP1 | 17 | 60218000 | 19 | 617205 | -10.3 | 9.77E-25 | -11.8 | 2.93E-32 | -15.6 | 5.18E-55 | 5.18E-55 | 0.743 | CM |
| ENSG00000223940.1 | KRT8P8 | 23 | 151648049 | 12 | 53295917 | -10.6 | 2.14E-26 | -11.3 | 1.26E-29 | -15.5 | 2.97E-54 | 2.97E-54 | 0.868 | CM |
| ENSG00000225178.4 | RPSAP56 | 16 | 73975423 | 3 | 39428241 | NA | NA | 15.5 | 3.84E-54 | NA | NA | 3.84E-54 | 0.442 | CM |
| ENSG00000197582.5 | GPX1P1 | 23 | 13397459 | 3 | 49443372 | 6.0 | 1.57E-09 | 15.4 | 7.99E-54 | 15.2 | 4.04E-52 | 7.99E-54 | 0.637 | CM |
| ENSG00000261377.1 | RP11-578F21.12 | 15 | 29034810 | 3 | 33902722 | 11.4 | 5.52E-30 | 10.0 | 1.14E-23 | 15.1 | 9.48E-52 | 9.48E-52 | 0.468 | CM |
| ENSG00000214263.2 | RPSAP53 | 13 | 67841838 | 3 | 39458770 | 15.0 | 4.79E-51 | NA | NA | NA | NA | 4.79E-51 | 0.758 | CM |
| ENSG00000268568.1 | AC007228.9 | 19 | 57185269 | 22 | 41817306 | -10.0 | 1.07E-23 | -10.9 | 6.93E-28 | -14.8 | 8.54E-50 | 8.54E-50 | 0.671 | CM |
| ENSG00000227615.1 | RP11-864N7.2 | 11 | 74457159 | 6 | 1.33E+08 | 14.7 | 3.15E-49 | NA | NA | NA | NA | 3.15E-49 | 0.657 | CM |
| ENSG00000240668.1 | KRT8P36 | 3 | 137821759 | 12 | 53295917 | -8.1 | 7.42E-16 | -12.5 | 4.96E-36 | -14.6 | 4.79E-48 | 4.79E-48 | 0.862 | CM |
| ENSG00000235655.2 | H3F3AP4 | 2 | 175584505 | 1 | 2.26E+08 | -8.0 | 9.44E-16 | -12.2 | 2.34E-34 | -14.3 | 1.55E-46 | 1.55E-46 | 0.277 | CM |
| ENSG00000227077.2 | AC107983.4 | 17 | 18476045 | 19 | 8394092 | 14.1 | 2.51E-45 | NA | NA | NA | NA | 2.51E-45 | 0.340 | CM |
| ENSG00000257513.3 | NPIPB1P | 18 | 11639698 | 16 | 70088532 | 8.0 | 8.50E-16 | 11.6 | 3.90E-31 | 13.9 | 6.73E-44 | 6.73E-44 | 0.349 | CM |
| ENSG00000180574.3 | EIF2S3L | 12 | 10658201 | 23 | 24096220 | 8.4 | 4.43E-17 | 11.0 | 4.70E-28 | 13.7 | 9.45E-43 | 9.45E-43 | 0.864 | CM |
| ENSG00000250539.1 | KRT8P33 | 5 | 122738038 | 12 | 53294381 | -4.1 | 3.49E-05 | -12.6 | 3.79E-36 | -11.8 | 3.75E-32 | 3.79E-36 | 0.780 | CM |
| ENSG00000233426.2 | EIF3FP3 | 2 | 58478575 | 11 | 8015651 | -9.2 | 2.59E-20 | -8.2 | 2.76E-16 | -12.3 | 7.39E-35 | 7.39E-35 | 0.719 | CM |
| ENSG00000256356.1 | HSPA8P5 | 12 | 4206617 | 11 | 1.23E+08 | 12.0 | 2.07E-33 | NA | NA | NA | NA | 2.07E-33 | 0.882 | CM |
| ENSG00000255815.2 | KRT8P11 | 9 | 102067394 | 12 | 53294381 | -9.5 | 1.37E-21 | -7.5 | 9.25E-14 | -12.0 | 2.88E-33 | 2.88E-33 | 0.866 | CM |
| ENSG00000217835.3 | RP6-159A1.2 | 23 | 65177038 | 9 | 6728376 | NA | NA | -12.0 | 3.80E-33 | NA | NA | 3.80E-33 | 0.619 | CM |
| ENSG00000261512.2 | RP11-46D6.1 | 16 | 46658363 | 3 | 1.29E+08 | -6.0 | 1.91E-09 | -11.0 | 6.37E-28 | -12.0 | 3.91E-33 | 3.91E-33 | 0.479 | CM |
| ENSG00000186940.6 | CHCHD2P9 | 9 | 82006219 | 7 | 56166087 | -12.0 | 4.91E-33 | NA | NA | NA | NA | 4.91E-33 | 0.691 | CM |
| ENSG00000189343.6 | RPS2P46 | 17 | 19350169 | 16 | 2014834 | 11.8 | 4.43E-32 | 4.4 | 1.02E-05 | 11.5 | 2.16E-30 | 4.43E-32 | 0.253 | CM |
| ENSG00000214016.3 | RP6-105D16.1 | 23 | 44600333 | 3 | 39433803 | -5.7 | 1.24E-08 | 11.8 | 6.04E-32 | 4.3 | 1.78E-05 | 6.04E-32 | 0.306 | CM |
| ENSG00000227018.1 | IL6STP1 | 17 | 15686602 | 5 | 55266512 | 11.5 | 1.17E-30 | NA | NA | NA | NA | 1.17E-30 | 0.873 | CM |
| ENSG00000235508.2 | RPS2P7 | 20 | 44270009 | 16 | 2011126 | NA | NA | -11.5 | 2.08E-30 | NA | NA | 2.08E-30 | 0.324 | CM |
| ENSG00000224520.2 | KRT8P45 | 1 | 157043049 | 12 | 53294381 | NA | NA | -11.4 | 5.55E-30 | NA | NA | 5.55E-30 | 0.729 | CM |
| ENSG00000187534.5 | CTC-471F3.4 | 19 | 40449634 | 12 | 53840385 | 11.3 | 1.04E-29 | NA | NA | NA | NA | 1.04E-29 | 0.586 | CM |
| ENSG00000188933.10 | USP32P1 | 17 | 16689803 | 23 | NA | NA | NA | 11.2 | 2.35E-29 | NA | NA | 2.35E-29 | 0.359 | CM |
| ENSG00000235559.1 | NOL5BP | 6 | 28751781 | 20 | 2650232 | 11.0 | 2.97E-28 | NA | NA | NA | NA | 2.97E-28 | 0.686 | CM |
| ENSG00000170160.12 | CCDC144A | 17 | 16592851 | 23 | NA | NA | NA | 11.0 | 4.16E-28 | NA | NA | 4.16E-28 | 0.316 | CM |
| ENSG00000215006.4 | CHCHD2P2 | 5 | 68629756 | 7 | 56071984 | -10.9 | 8.01E-28 | NA | NA | NA | NA | 8.01E-28 | 0.726 | CM |
| ENSG00000228674.3 | CTB-147C22.3 | 19 | 51430831 | 7 | 44839429 | 10.8 | 2.71E-27 | NA | NA | NA | NA | 2.71E-27 | 0.851 | CM |
| ENSG00000233913.6 | CTC-575D19.1 | 5 | 168043317 | 23 | 1.54E+08 | NA | NA | 10.8 | 3.74E-27 | NA | NA | 3.74E-27 | 0.367 | CM |
| ENSG00000188801.8 | ZNF322P1 | 9 | 99961910 | 6 | 26665835 | -10.7 | 1.16E-26 | NA | NA | NA | NA | 1.16E-26 | 0.514 | CM |
| ENSG00000261172.1 | RP11-356C4.5 | 16 | 90234633 | 20 | 63244 | NA | NA | 10.7 | 1.37E-26 | NA | NA | 1.37E-26 | 0.169 | CM |
| ENSG00000214249.3 | CTAGE11P | 13 | 75814432 | 14 | 39774149 | -10.6 | 1.91E-26 | NA | NA | NA | NA | 1.91E-26 | 0.618 | CM |
| ENSG00000236816.2 | ANKRD20A7P | 9 | 44118444 | 23 | NA | NA | NA | 10.5 | 7.83E-26 | NA | NA | 7.83E-26 | 0.282 | CM |
| ENSG00000228305.1 | AC016734.2 | 2 | 63849965 | 5 | 1.77E+08 | -9.7 | 4.66E-22 | -4.7 | 2.75E-06 | -10.1 | 3.57E-24 | 3.57E-24 | 0.563 | CM |
| ENSG00000214787.4 | MS4A4E | 11 | 60010561 | 20 | 37009466 | NA | NA | -10.0 | 1.63E-23 | NA | NA | 1.63E-23 | 0.448 | CM |
| ENSG00000263535.1 | AK4P1 | 17 | 29672539 | 1 | 65690461 | NA | NA | 10.0 | 1.77E-23 | NA | NA | 1.77E-23 | 0.371 | CM |
| ENSG00000267809.1 | NDUFV2P1 | 19 | 53728132 | 18 | 9177431 | -7.2 | 6.05E-13 | -6.9 | 5.86E-12 | -10.0 | 2.33E-23 | 2.33E-23 | 0.795 | CM |
| ENSG00000147274.10 | RBMX | 23 | 135962923 | 9 | 6728376 | NA | NA | -9.9 | 2.81E-23 | NA | NA | 2.81E-23 | 0.724 | CM |
| ENSG00000249465.1 | RBMXP4 | 4 | 110268615 | 9 | 6722212 | NA | NA | -9.9 | 2.92E-23 | NA | NA | 2.92E-23 | 0.839 | CM |
| ENSG00000260528.2 | FAM157C | 16 | 90168679 | 20 | 63244 | 4.2 | 2.42E-05 | 9.8 | 1.70E-22 | 9.9 | 4.81E-23 | 4.81E-23 | 0.158 | CM |
| ENSG00000196656.6 | AC004057.1 | 4 | 114138326 | 12 | 56401085 | 6.9 | 4.80E-12 | 6.7 | 2.12E-11 | 9.6 | 6.40E-22 | 6.40E-22 | 0.186 | CM |
| ENSG00000139239.6 | RPL14P1 | 12 | 63359095 | 3 | 40498845 | -9.6 | 8.51E-22 | NA | NA | NA | NA | 8.51E-22 | 0.721 | CM |
| ENSG00000261172.1 | RP11-356C4.5 | 16 | 90234633 | 23 | 1.55E+08 | NA | NA | 9.6 | 8.97E-22 | NA | NA | 8.97E-22 | 0.169 | CM |
| ENSG00000230076.1 | AC016708.2 | 2 | 215711852 | 23 | 1.54E+08 | NA | NA | 9.5 | 1.41E-21 | NA | NA | 1.41E-21 | 0.295 | CM |
| ENSG00000180015.11 | RP11-756P10.3 | 4 | 189659506 | 6 | 1.53E+08 | -7.2 | 4.80E-13 | -6.1 | 1.09E-09 | -9.4 | 4.38E-21 | 4.38E-21 | 0.667 | CM |
| ENSG00000213574.2 | LDHAP5 | 10 | 120692807 | 11 | 18428264 | -9.4 | 7.82E-21 | NA | NA | NA | NA | 7.82E-21 | 0.865 | CM |
| ENSG00000253954.2 | HMGN1P38 | 15 | 93254977 | 21 | 40717478 | 9.3 | 9.41E-21 | NA | NA | NA | NA | 9.41E-21 | 0.390 | CM |
| ENSG00000197258.5 | EIF4BP6 | 7 | 104308196 | 12 | 53423711 | NA | NA | -9.3 | 1.24E-20 | NA | NA | 1.24E-20 | 0.478 | CM |
| ENSG00000241860.2 | RP11-34P13.13 | 1 | 173862 | 16 | 90244341 | NA | NA | 9.2 | 5.47E-20 | NA | NA | 5.47E-20 | 0.093 | CM |
| ENSG00000260528.2 | FAM157C | 16 | 90168679 | 23 | NA | NA | NA | 8.9 | 4.59E-19 | NA | NA | 4.59E-19 | 0.158 | CM |
| ENSG00000213492.2 | NT5C3AP1 | 4 | 118497329 | 7 | 33098763 | NA | NA | -8.9 | 6.80E-19 | NA | NA | 6.80E-19 | 0.811 | CM |
| ENSG00000157399.10 | ARSE | 23 | 2886286 | 16 | 79541257 | -5.9 | 3.54E-09 | -6.6 | 3.25E-11 | -8.9 | 7.56E-19 | 7.56E-19 | 0.876 | CM |
| ENSG00000259480.1 | RP11-26F2.1 | 15 | 23145997 | 3 | 33909611 | 8.7 | 2.80E-18 | NA | NA | NA | NA | 2.80E-18 | 0.473 | CM |
| ENSG00000164669.8 | INTS4L1 | 7 | 64601603 | 11 | 77639511 | 8.7 | 4.52E-18 | NA | NA | NA | NA | 4.52E-18 | 0.756 | CM |
| ENSG00000242766.1 | IGKV1D-17 | 2 | 90121477 | 23 | NA | NA | NA | 8.6 | 5.62E-18 | NA | NA | 5.62E-18 | 0.414 | CM |
| ENSG00000232573.1 | RPL3P4 | 14 | 99439638 | 22 | 39706598 | NA | NA | 8.6 | 8.96E-18 | NA | NA | 8.96E-18 | 0.513 | CM |
| ENSG00000238072.1 | RP11-305M3.2 | 7 | 129050211 | 9 | 1.16E+08 | 4.2 | 2.94E-05 | 7.9 | 1.88E-15 | 8.6 | 9.88E-18 | 9.88E-18 | 0.682 | CM |
| ENSG00000220378.3 | KRT8P42 | 6 | 134619833 | 12 | 53300203 | 8.6 | 1.02E-17 | NA | NA | NA | NA | 1.02E-17 | 0.894 | CM |
| ENSG00000234176.1 | HSPA8P1 | 23 | 120338868 | 11 | 1.23E+08 | 8.5 | 1.24E-17 | NA | NA | NA | NA | 1.24E-17 | 0.868 | CM |
| ENSG00000170165.5 | CR848007.2 | 9 | 44069881 | 12 | 54478114 | NA | NA | 8.5 | 1.27E-17 | NA | NA | 1.27E-17 | 0.201 | CM |
| ENSG00000230734.1 | RPL10P3 | 9 | 119943344 | 23 | 1.54E+08 | -8.5 | 1.30E-17 | NA | NA | NA | NA | 1.30E-17 | 0.692 | CM |
| ENSG00000236816.2 | ANKRD20A7P | 9 | 44118444 | 12 | 54478114 | NA | NA | 8.5 | 2.89E-17 | NA | NA | 2.89E-17 | 0.282 | CM |
| ENSG00000253497.1 | IGKV1-13 | 2 | 89345963 | 9 | 42600882 | NA | NA | 8.4 | 3.81E-17 | NA | NA | 3.81E-17 | 0.349 | CM |
| ENSG00000253497.1 | IGKV1-13 | 2 | 89345963 | 10 | 42760666 | NA | NA | 8.3 | 9.33E-17 | NA | NA | 9.33E-17 | 0.349 | CM |
| ENSG00000170165.5 | CR848007.2 | 9 | 44069881 | 1 | 1.04E+08 | NA | NA | 8.3 | 9.79E-17 | NA | NA | 9.79E-17 | 0.201 | CM |
| ENSG00000241860.2 | RP11-34P13.13 | 1 | 173862 | 20 | 63244 | NA | NA | 8.3 | 1.02E-16 | NA | NA | 1.02E-16 | 0.093 | CM |
| ENSG00000157399.10 | ARSE | 23 | 2886286 | 17 | 54869495 | -7.6 | 2.77E-14 | -4.0 | 5.18E-05 | -8.2 | 1.70E-16 | 1.70E-16 | 0.876 | CM |
| ENSG00000224546.2 | EIF4BP3 | 9 | 98908289 | 12 | 53423711 | NA | NA | -8.2 | 2.04E-16 | NA | NA | 2.04E-16 | 0.483 | CM |
| ENSG00000138413.9 | IDH1 | 2 | 209130798 | 3 | 1.46E+08 | 8.2 | 2.17E-16 | NA | NA | NA | NA | 2.17E-16 | 0.886 | CM |
| ENSG00000214975.4 | PPIAP29 | 6 | 24976647 | 7 | 44836314 | 8.2 | 2.50E-16 | NA | NA | NA | NA | 2.50E-16 | 0.356 | CM |
| ENSG00000236816.2 | ANKRD20A7P | 9 | 44118444 | 1 | 1.04E+08 | NA | NA | 8.2 | 2.87E-16 | NA | NA | 2.87E-16 | 0.282 | CM |
| ENSG00000170165.5 | CR848007.2 | 9 | 44069881 | 23 | NA | NA | NA | 8.2 | 3.59E-16 | NA | NA | 3.59E-16 | 0.201 | CM |
| ENSG00000241860.2 | RP11-34P13.13 | 1 | 173862 | 23 | NA | NA | NA | 8.1 | 4.08E-16 | NA | NA | 4.08E-16 | 0.093 | CM |
| ENSG00000178715.7 | RP11-169K16.8 | 1 | 16154724 | 19 | 39928107 | 4.8 | 1.30E-06 | 6.6 | 3.37E-11 | 8.1 | 5.06E-16 | 5.06E-16 | 0.895 | CM |
| ENSG00000260923.1 | AC137934.1 | 16 | 90252405 | 2 | 2.43E+08 | NA | NA | -8.1 | 5.96E-16 | NA | NA | 5.96E-16 | 0.173 | CM |
| ENSG00000157399.10 | ARSE | 23 | 2886286 | 15 | 97721414 | -4.6 | 3.81E-06 | -6.8 | 9.38E-12 | -8.1 | 6.10E-16 | 6.10E-16 | 0.876 | CM |
| ENSG00000233225.2 | AC004987.9 | 7 | 39872813 | 1 | 54742005 | 5.0 | 5.98E-07 | 6.4 | 1.21E-10 | 8.1 | 6.36E-16 | 6.36E-16 | 0.750 | CM |
| ENSG00000157399.10 | ARSE | 23 | 2886286 | 2 | 2.22E+08 | -5.4 | 8.70E-08 | -6.0 | 1.59E-09 | -8.1 | 8.15E-16 | 8.15E-16 | 0.876 | CM |
| ENSG00000250966.2 | RP11-402J6.3 | 4 | 113486144 | 1 | 2.26E+08 | NA | NA | 8.0 | 8.36E-16 | NA | NA | 8.36E-16 | 0.304 | CM |
| ENSG00000157399.10 | ARSE | 23 | 2886286 | 1 | 84689813 | -8.0 | 8.56E-16 | NA | NA | NA | NA | 8.56E-16 | 0.876 | CM |
| ENSG00000157399.10 | ARSE | 23 | 2886286 | 20 | 53603833 | -8.0 | 1.42E-15 | NA | NA | NA | NA | 1.42E-15 | 0.876 | CM |
| ENSG00000253525.1 | CTD-2114J12.1 | 8 | 92169513 | 3 | 1.49E+08 | -7.0 | 2.95E-12 | -4.3 | 1.82E-05 | -8.0 | 1.64E-15 | 1.64E-15 | 0.826 | CM |
| ENSG00000138413.9 | IDH1 | 2 | 209130798 | 5 | 92808367 | 7.9 | 1.97E-15 | NA | NA | NA | NA | 1.97E-15 | 0.886 | CM |
| ENSG00000138413.9 | IDH1 | 2 | 209130798 | 12 | 1.29E+08 | 7.9 | 2.94E-15 | NA | NA | NA | NA | 2.94E-15 | 0.886 | CM |
| ENSG00000172785.14 | CBWD1 | 9 | 188979 | 2 | 1.14E+08 | NA | NA | 7.9 | 3.26E-15 | NA | NA | 3.26E-15 | 0.230 | CM |
| ENSG00000182484.10 | WASH6P | 23 | 155250491 | 15 | 1.02E+08 | NA | NA | 7.9 | 3.40E-15 | NA | NA | 3.40E-15 | 0.155 | CM |
| ENSG00000232573.1 | RPL3P4 | 14 | 99439638 | 1 | 1.66E+08 | NA | NA | 7.9 | 3.45E-15 | NA | NA | 3.45E-15 | 0.513 | CM |
| ENSG00000157399.10 | ARSE | 23 | 2886286 | 9 | 1.05E+08 | -7.9 | 3.99E-15 | NA | NA | NA | NA | 3.99E-15 | 0.876 | CM |
| ENSG00000157399.10 | ARSE | 23 | 2886286 | 4 | 36312243 | -7.9 | 4.07E-15 | NA | NA | NA | NA | 4.07E-15 | 0.876 | CM |
| ENSG00000138413.9 | IDH1 | 2 | 209130798 | 23 | 64453580 | 7.8 | 5.77E-15 | NA | NA | NA | NA | 5.77E-15 | 0.886 | CM |
| ENSG00000232573.1 | RPL3P4 | 14 | 99439638 | 4 | 1.07E+08 | NA | NA | 7.8 | 6.26E-15 | NA | NA | 6.26E-15 | 0.513 | CM |
| ENSG00000232573.1 | RPL3P4 | 14 | 99439638 | 3 | 52053393 | NA | NA | 7.8 | 6.26E-15 | NA | NA | 6.26E-15 | 0.513 | CM |
| ENSG00000157399.10 | ARSE | 23 | 2886286 | 14 | 69320202 | -7.8 | 7.41E-15 | NA | NA | NA | NA | 7.41E-15 | 0.876 | CM |
| ENSG00000157399.10 | ARSE | 23 | 2886286 | 3 | 1.57E+08 | -7.7 | 1.05E-14 | NA | NA | NA | NA | 1.05E-14 | 0.876 | CM |
| ENSG00000157399.10 | ARSE | 23 | 2886286 | 13 | 1.09E+08 | -7.7 | 1.12E-14 | NA | NA | NA | NA | 1.12E-14 | 0.876 | CM |
| ENSG00000230880.2 | RP11-417J8.3 | 1 | 142618769 | 4 | 49061848 | 7.7 | 1.58E-14 | NA | NA | NA | NA | 1.58E-14 | 0.351 | CM |
| ENSG00000157399.10 | ARSE | 23 | 2886286 | 11 | 1.1E+08 | -7.6 | 2.10E-14 | NA | NA | NA | NA | 2.10E-14 | 0.876 | CM |
| ENSG00000157326.14 | DHRS4 | 14 | 24424244 | 7 | 12172674 | NA | NA | 7.6 | 2.14E-14 | NA | NA | 2.14E-14 | 0.478 | CM |
| ENSG00000171943.7 | SRGAP2C | 1 | 121107124 | 3 | 24100165 | NA | NA | 7.6 | 3.89E-14 | NA | NA | 3.89E-14 | 0.496 | CM |
| ENSG00000157326.14 | DHRS4 | 14 | 24424244 | 1 | 2.29E+08 | NA | NA | 7.6 | 3.89E-14 | NA | NA | 3.89E-14 | 0.478 | CM |
| ENSG00000243517.1 | RP11-627K11.1 | 12 | 31405531 | 8 | 1.33E+08 | -7.5 | 4.92E-14 | NA | NA | NA | NA | 4.92E-14 | 0.523 | CM |
| ENSG00000157399.10 | ARSE | 23 | 2886286 | 12 | 1.33E+08 | -5.2 | 2.09E-07 | -5.4 | 8.76E-08 | -7.5 | 9.06E-14 | 9.06E-14 | 0.876 | CM |
| ENSG00000226396.1 | RPS14P3 | 1 | 19934608 | 5 | 1.5E+08 | NA | NA | 7.5 | 9.19E-14 | NA | NA | 9.19E-14 | 0.846 | CM |
| ENSG00000232573.1 | RPL3P4 | 14 | 99439638 | 15 | 89864614 | NA | NA | 7.4 | 1.01E-13 | NA | NA | 1.01E-13 | 0.513 | CM |
| ENSG00000232573.1 | RPL3P4 | 14 | 99439638 | 13 | 81765636 | NA | NA | 7.4 | 1.01E-13 | NA | NA | 1.01E-13 | 0.513 | CM |
| ENSG00000230635.1 | CYP4F60P | 9 | 44020591 | 12 | 54478114 | NA | NA | 7.4 | 1.11E-13 | NA | NA | 1.11E-13 | 0.198 | CM |
| ENSG00000160072.15 | ATAD3B | 1 | 1407143 | 5 | 1.16E+08 | NA | NA | -7.4 | 1.12E-13 | NA | NA | 1.12E-13 | 0.755 | CM |
| ENSG00000157399.10 | ARSE | 23 | 2886286 | 8 | 80357822 | -7.4 | 1.23E-13 | NA | NA | NA | NA | 1.23E-13 | 0.876 | CM |
| ENSG00000232573.1 | RPL3P4 | 14 | 99439638 | 8 | 1.03E+08 | NA | NA | 7.4 | 1.31E-13 | NA | NA | 1.31E-13 | 0.513 | CM |
| ENSG00000232573.1 | RPL3P4 | 14 | 99439638 | 18 | 30413440 | NA | NA | 7.4 | 1.31E-13 | NA | NA | 1.31E-13 | 0.513 | CM |
| ENSG00000226210.3 | ABC7-42389800N19.1 | 12 | 73725 | 9 | 51868 | NA | NA | 7.4 | 1.40E-13 | NA | NA | 1.40E-13 | 0.208 | CM |
| ENSG00000206082.8 | LINC01002 | 19 | 237967 | 16 | 90256928 | NA | NA | 7.4 | 1.54E-13 | NA | NA | 1.54E-13 | 0.101 | CM |
| ENSG00000232573.1 | RPL3P4 | 14 | 99439638 | 9 | 660320 | NA | NA | 7.3 | 2.08E-13 | NA | NA | 2.08E-13 | 0.513 | CM |
| ENSG00000144820.3 | GPR128 | 3 | 100328433 | 6 | 1.43E+08 | 8.2 | 2.40E-16 | NA | NA | NA | NA | 2.40E-16 | 1.000 | MH |
| ENSG00000144820.3 | GPR128 | 3 | 100328433 | 8 | 6165636 | 7.9 | 1.90E-15 | NA | NA | NA | NA | 1.90E-15 | 1.000 | MH |
| ENSG00000144820.3 | GPR128 | 3 | 100328433 | 23 | 86752630 | 7.5 | 4.87E-14 | NA | NA | NA | NA | 4.87E-14 | 1.000 | MH |
| ENSG00000144820.3 | GPR128 | 3 | 100328433 | 1 | 1.16E+08 | 7.4 | 1.19E-13 | NA | NA | NA | NA | 1.19E-13 | 1.000 | MH |
| ENSG00000213058.3 | RP4-765C7.2 | 1 | 178380751 | 5 | 1.5E+08 | 10.7 | 1.30E-26 | 14.2 | 6.39E-46 | 17.6 | 2.10E-69 | 2.10E-69 | 0.917 | PA |
| ENSG00000236496.2 | RP11-255J3.2 | 9 | 2876427 | 17 | 7208197 | -7.2 | 7.70E-13 | -11.6 | 3.46E-31 | -13.3 | 3.01E-40 | 3.01E-40 | 0.970 | PA |
| ENSG00000250982.2 | RP11-159J3.1 | 16 | 28251989 | 12 | 6646320 | 11.8 | 2.51E-32 | NA | NA | NA | NA | 2.51E-32 | 0.988 | PA |
| ENSG00000261770.1 | CTC-459F4.1 | 19 | 28251757 | 5 | 49407265 | NA | NA | 8.8 | 1.73E-18 | NA | NA | 1.73E-18 | 0.972 | PA |
| ENSG00000262881.1 | RP11-669E14.4 | 17 | 43988145 | 12 | 74560707 | NA | NA | -7.6 | 3.60E-14 | NA | NA | 3.60E-14 | 1.000 | PA |
| ENSG00000121089.4 | NACA3P | 4 | 165864442 | 12 | 57106660 | -13.2 | 4.62E-40 | -10.9 | 1.99E-27 | -17.0 | 4.13E-65 | 4.13E-65 | 0.969 | PG |
| ENSG00000236090.2 | LDHAP3 | 2 | 42046887 | 11 | 18414620 | -14.4 | 8.97E-47 | -7.1 | 1.35E-12 | -15.2 | 5.74E-52 | 5.74E-52 | 0.918 | PG |
| ENSG00000233606.1 | OR6C66P | 12 | 55782229 | 2 | 1.79E+08 | 14.4 | 1.03E-46 | NA | NA | NA | NA | 1.03E-46 | 1.000 | PG |
| ENSG00000225774.1 | SIRPAP1 | 22 | 30938523 | 20 | 1889858 | 7.2 | 5.27E-13 | 12.6 | 1.75E-36 | 14.0 | 1.11E-44 | 1.11E-44 | 0.932 | PG |
| ENSG00000235847.2 | LDHAP7 | 2 | 85005347 | 11 | 18420246 | -11.5 | 1.02E-30 | -6.7 | 2.42E-11 | -12.9 | 6.66E-38 | 6.66E-38 | 0.928 | PG |
| ENSG00000226666.1 | HSPA9P1 | 2 | 222826456 | 5 | 1.38E+08 | 12.9 | 6.70E-38 | NA | NA | NA | NA | 6.70E-38 | 0.945 | PG |
| ENSG00000227183.3 | HDGFP1 | 23 | 130780648 | 1 | 1.57E+08 | 11.9 | 8.56E-33 | NA | NA | NA | NA | 8.56E-33 | 0.972 | PG |
| ENSG00000241478.1 | HSPA8P9 | 3 | 137599137 | 11 | 1.23E+08 | 11.7 | 9.39E-32 | NA | NA | NA | NA | 9.39E-32 | 0.907 | PG |
| ENSG00000225308.2 | ASS1P11 | 7 | 21261047 | 9 | 1.33E+08 | 10.8 | 3.00E-27 | 5.8 | 7.95E-09 | 11.7 | 9.46E-32 | 9.46E-32 | 0.966 | PG |
| ENSG00000259500.1 | KRT8P24 | 15 | 49264000 | 12 | 53295917 | -11.3 | 2.09E-29 | NA | NA | NA | NA | 2.09E-29 | 0.976 | PG |
| ENSG00000236257.1 | EI24P2 | 1 | 158423988 | 11 | 1.25E+08 | 4.2 | 2.45E-05 | 11.2 | 6.21E-29 | 10.9 | 1.49E-27 | 6.21E-29 | 0.966 | PG |
| ENSG00000239830.1 | RPS4XP22 | 19 | 9794749 | 23 | 71493691 | -10.8 | 5.02E-27 | NA | NA | NA | NA | 5.02E-27 | 0.979 | PG |
| ENSG00000230849.2 | GOT2P2 | 1 | 173111489 | 16 | 58750604 | -9.2 | 4.75E-20 | NA | NA | NA | NA | 4.75E-20 | 0.968 | PG |
| ENSG00000233328.3 | PFN1P1 | 1 | 171640079 | 17 | 4849284 | NA | NA | 7.9 | 2.89E-15 | NA | NA | 2.89E-15 | 0.913 | PG |
| ENSG00000185684.8 | EP400NL | 12 | 132568828 | 22 | 40081605 | -7.7 | 1.05E-14 | NA | NA | NA | NA | 1.05E-14 | 0.916 | PG |

^a^ This and following four columns refer to the “target” gene influenced by the eQTL. ^b^ This and following column refer to the eQTL SNP variant. ^c^ z-statistic for association of reference SNP allele with expression of target gene. ^d^ cross-mappability score of target gene. ^e^ quality score assignment for association. HQ: high quality. CM: potentially spurious cross-mapping. MH: implausible “multiple hits,” potentially spurious. PA: poorly annotated target gene/mRNA. PG: target gene is a pseudogene.
