# Supplementary figures and images for: A resource for integrated genomic analysis of the human liver
Source: Sci Rep. 2022 Sep 7;12:15151. doi: 10.1038/s41598-022-18506-z (PMC9452507; doi:10.1038/s41598-022-18506-z)

### Individual SNP association, UNC liver

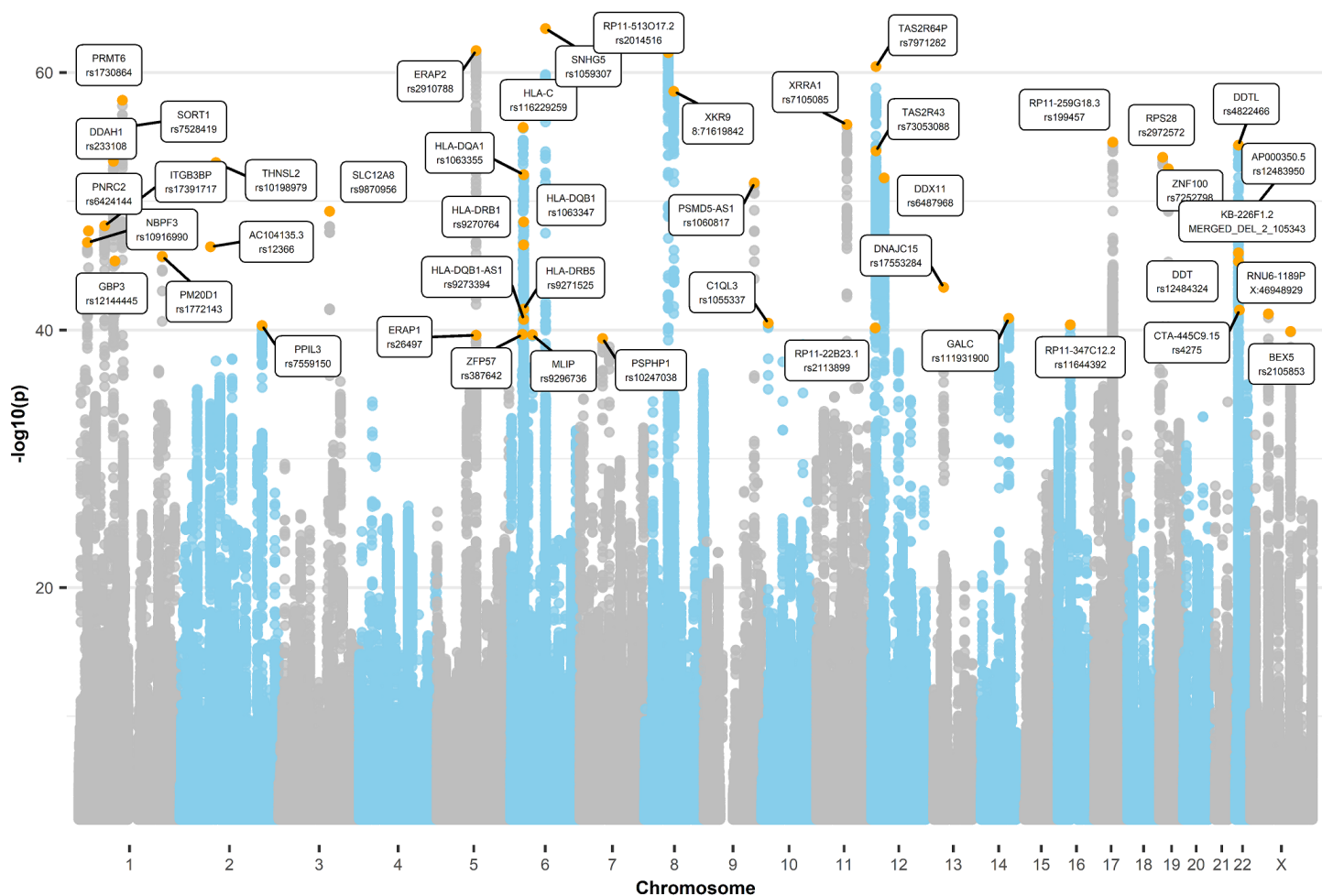

### Individual SNP association, GTEx liver

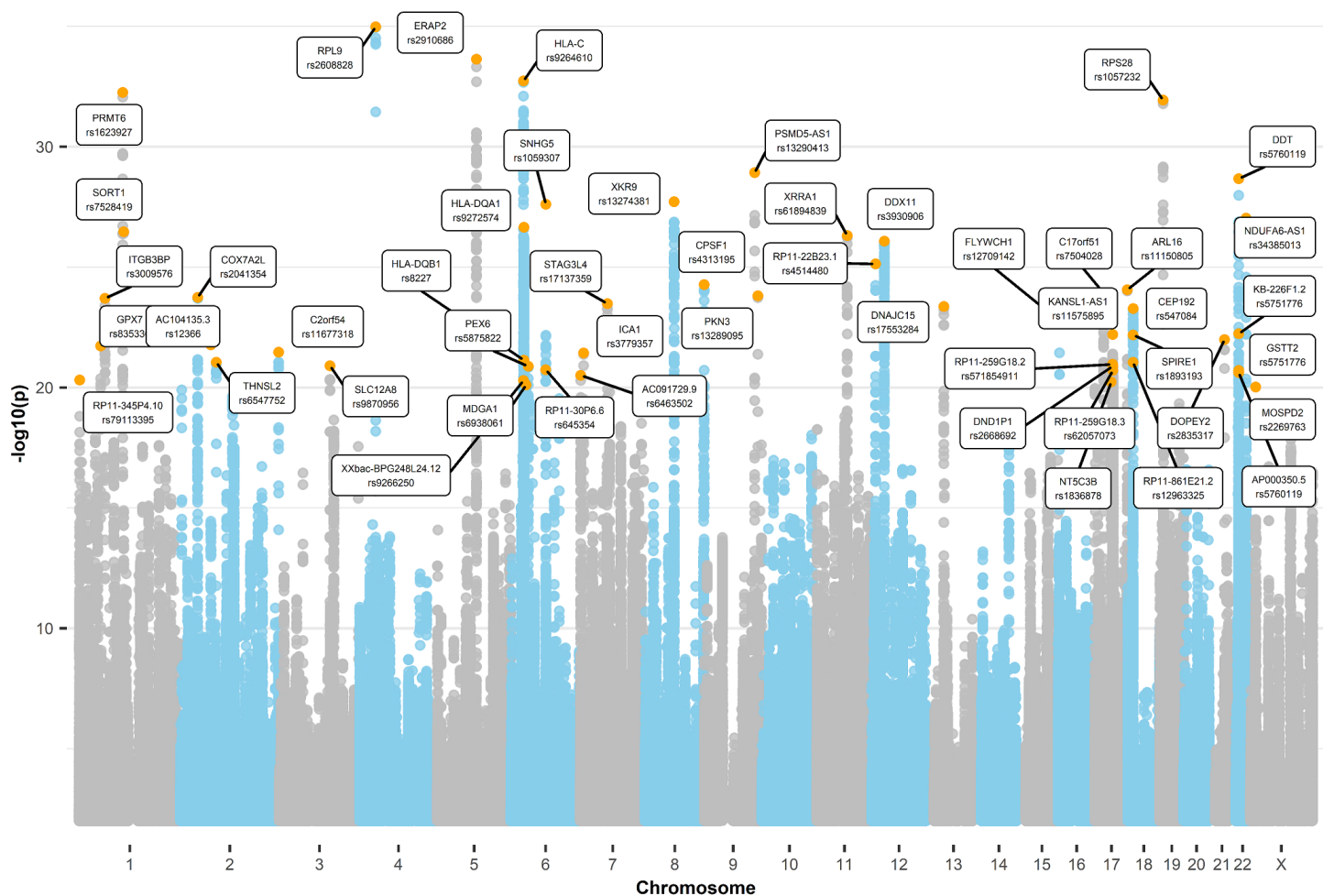

Supplement: Supplementary file 4 — Supplementary Figure 1. [file 41598_2022_18506_MOESM4_ESM.pdf]

eGene association, UNC Liver

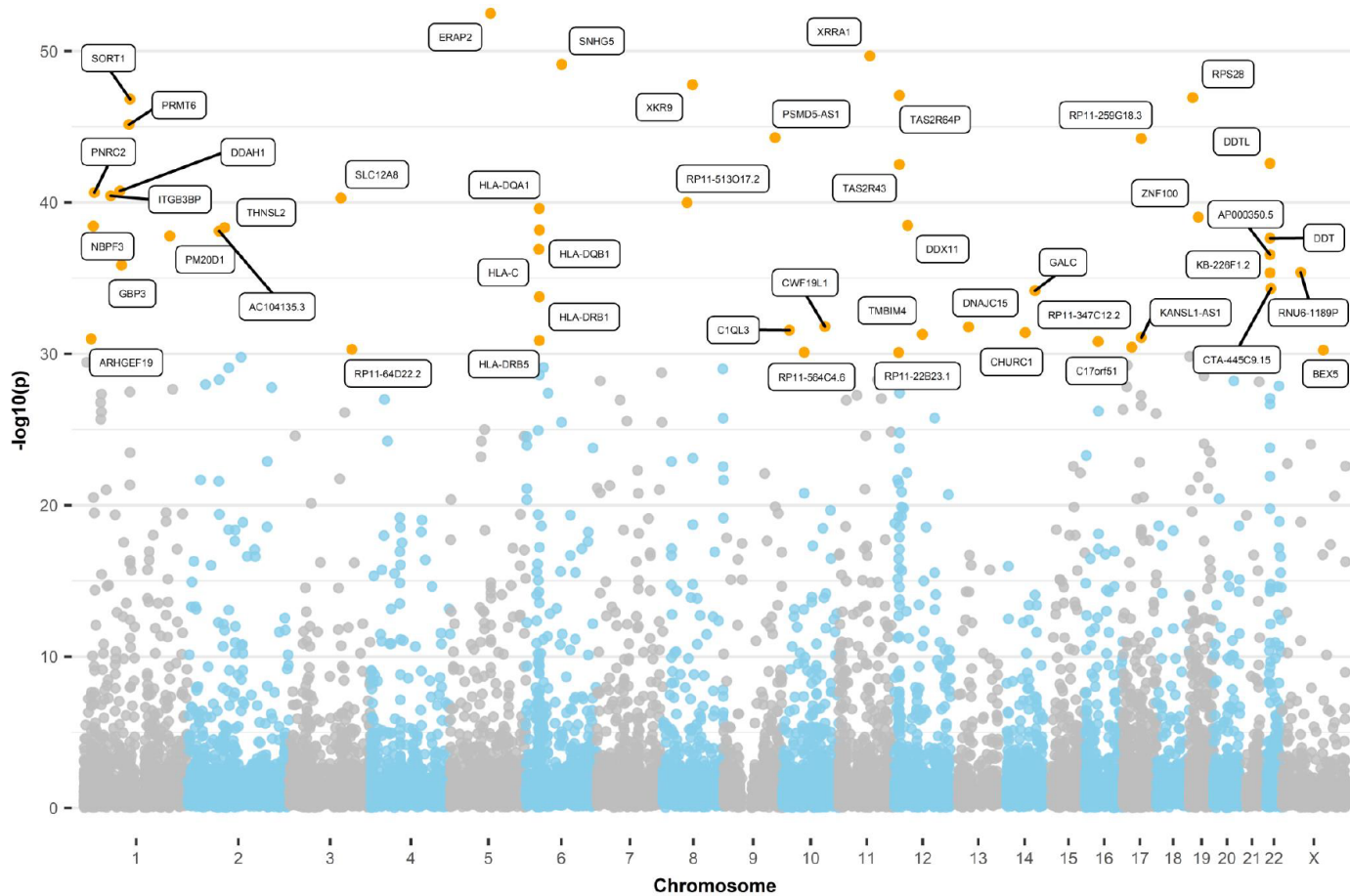

## eGene association, GTEx Liver

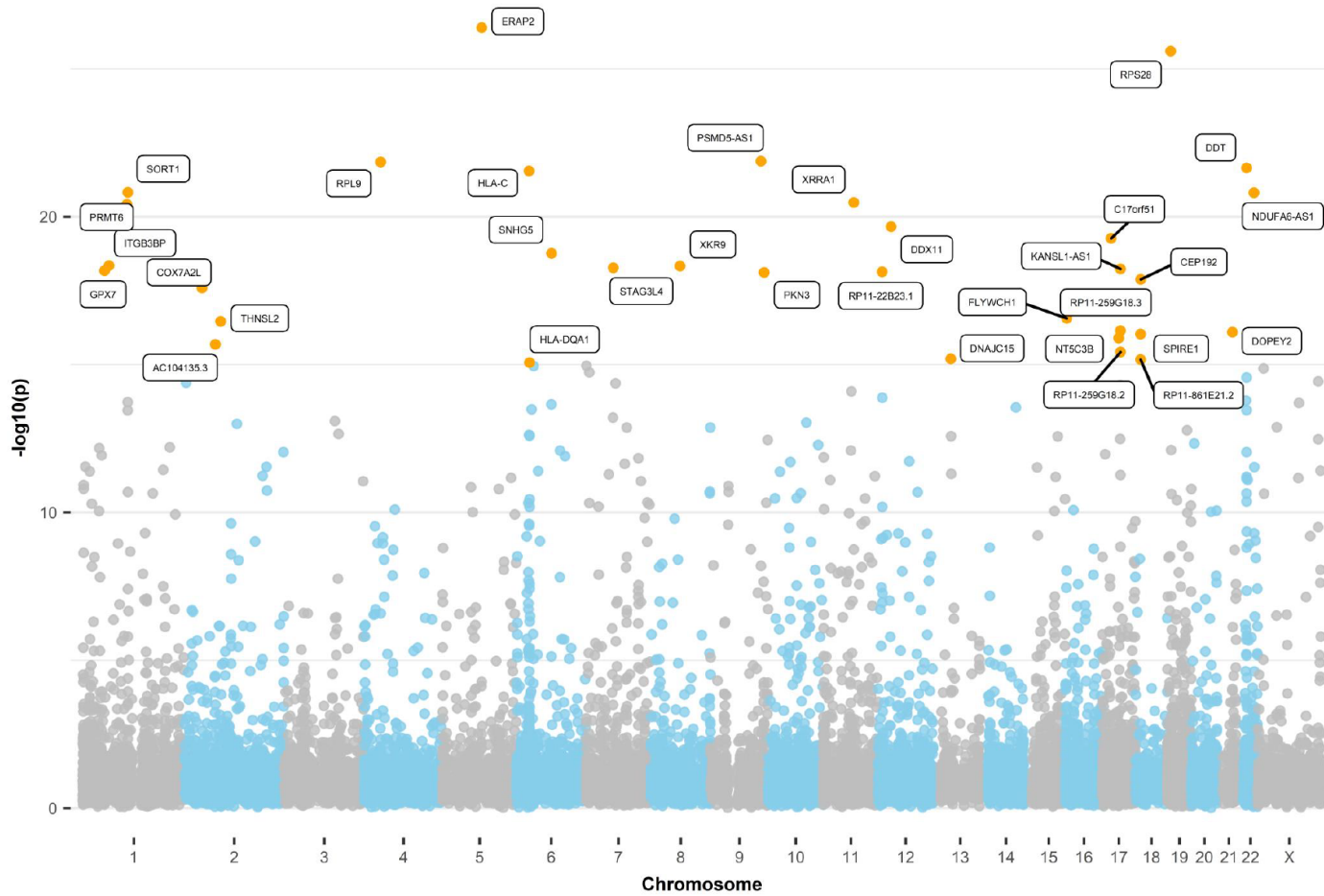

Supplement: Supplementary file 5 — Supplementary Figure 2. [file 41598_2022_18506_MOESM5_ESM.pdf]

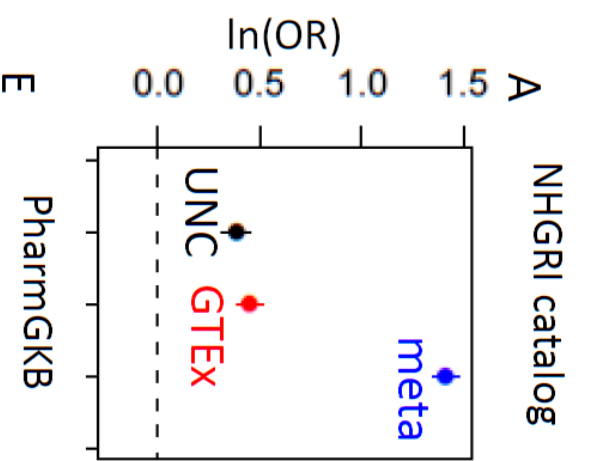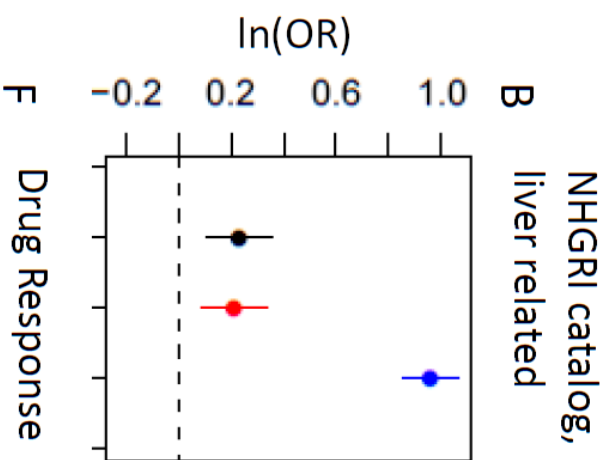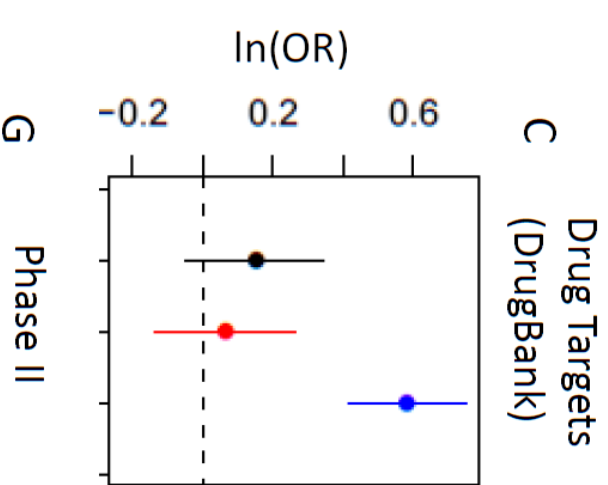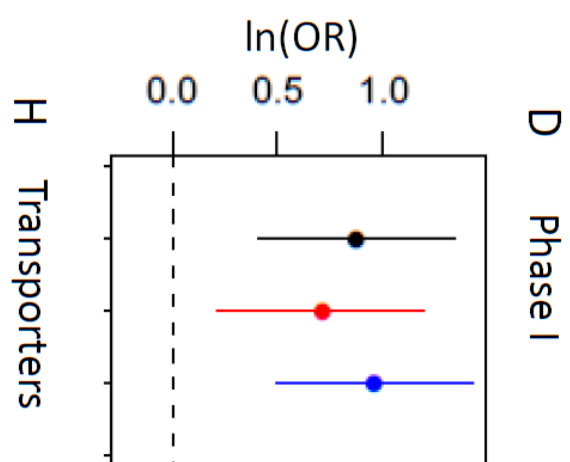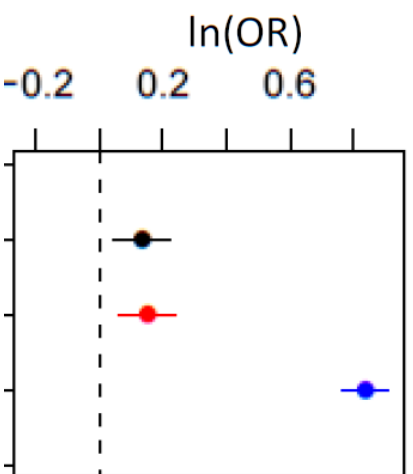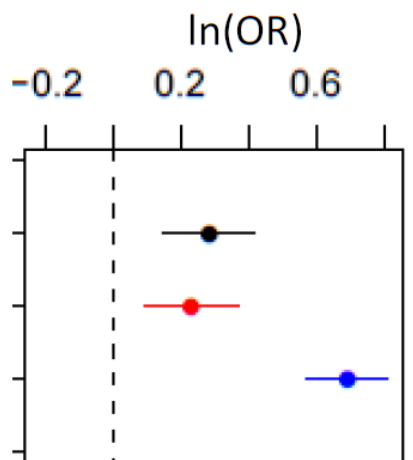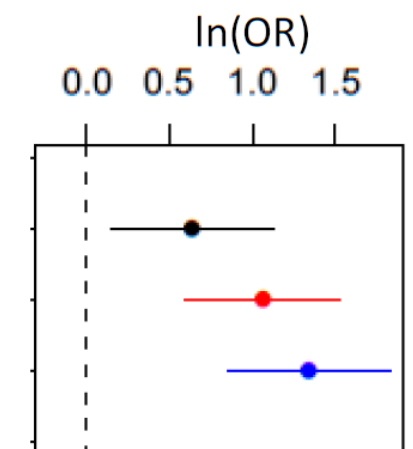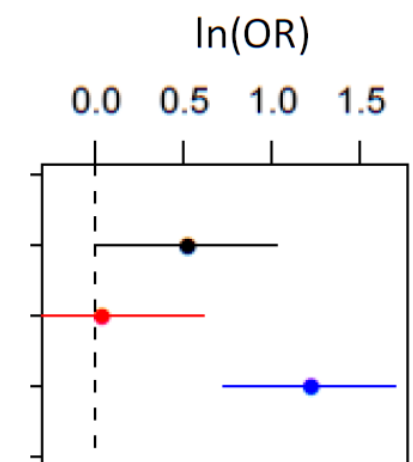

Supplement: Supplementary file 6 — Supplementary Figure 3. [file 41598_2022_18506_MOESM6_ESM.pdf]
